# Tumor-associated macrophage-mediated delivery of nano-photosensitizer enables light-induced metabolic programming for immuno-photodynamic therapy

**DOI:** 10.1093/procel/pwaf064

**Published:** 2025-08-20

**Authors:** Hong Deng, Huimin Wang, Yiyi Zhang, Runmeng Liu, Wei Hou, Lin Wang, Haiyan Xu, Weiqi Zhang

**Affiliations:** State Key Laboratory of Complex Severe and Rare Diseases, Institute of Basic Medical Sciences, Chinese Academy of Medical Sciences and Peking Union Medical College, Beijing 100005, China; Department of Biomedical Engineering, Institute of Basic Medical Sciences, Chinese Academy of Medical Sciences and Peking Union Medical College, Beijing 100005, China; State Key Laboratory of Complex Severe and Rare Diseases, Institute of Basic Medical Sciences, Chinese Academy of Medical Sciences and Peking Union Medical College, Beijing 100005, China; Department of Biomedical Engineering, Institute of Basic Medical Sciences, Chinese Academy of Medical Sciences and Peking Union Medical College, Beijing 100005, China; State Key Laboratory of Complex Severe and Rare Diseases, Institute of Basic Medical Sciences, Chinese Academy of Medical Sciences and Peking Union Medical College, Beijing 100005, China; Department of Biomedical Engineering, Institute of Basic Medical Sciences, Chinese Academy of Medical Sciences and Peking Union Medical College, Beijing 100005, China; State Key Laboratory of Complex Severe and Rare Diseases, Institute of Basic Medical Sciences, Chinese Academy of Medical Sciences and Peking Union Medical College, Beijing 100005, China; Department of Biomedical Engineering, Institute of Basic Medical Sciences, Chinese Academy of Medical Sciences and Peking Union Medical College, Beijing 100005, China; State Key Laboratory of Complex Severe and Rare Diseases, Institute of Basic Medical Sciences, Chinese Academy of Medical Sciences and Peking Union Medical College, Beijing 100005, China; Department of Biomedical Engineering, Institute of Basic Medical Sciences, Chinese Academy of Medical Sciences and Peking Union Medical College, Beijing 100005, China; State Key Laboratory of Common Mechanism Research for Major Diseases, Institute of Basic Medical Sciences, Chinese Academy of Medical Sciences and Peking Union Medical College, Beijing 100005, China; Department of Biomedical Engineering, Institute of Basic Medical Sciences, Chinese Academy of Medical Sciences and Peking Union Medical College, Beijing 100005, China; State Key Laboratory of Complex Severe and Rare Diseases, Institute of Basic Medical Sciences, Chinese Academy of Medical Sciences and Peking Union Medical College, Beijing 100005, China; Department of Biomedical Engineering, Institute of Basic Medical Sciences, Chinese Academy of Medical Sciences and Peking Union Medical College, Beijing 100005, China

## Dear Editor,

Tumor-associated macrophages (TAMs) account for up to 50% of tumor mass and serve as key innate immune cells in the tumor microenvironment (Kzhyshkowska et al., 2024). TAMs are primarily M2-like phenotypes that contribute to tumor immunosuppression and promote immune escape by secreting anti-inflammatory factors, thereby supporting tumor growth, metastasis, and angiogenesis. TAMs can be reprogrammed into M1-like phenotypes by immunostimulatory signals, promoting pro-inflammatory cytokines such as TNF-α and IL-12 that suppress tumor growth. Growing evidence highlights TAM modulation as an effective anticancer approach by boosting immunotherapeutic responses, primarily through facilitating M2-to-M1 polarization (Mantovani et al., 2022). However, fully harnessing the therapeutic potential of TAMs for cancer therapy requires both precise targeting and functional reprogramming, which remains a significant challenge.

Photodynamic therapy (PDT) is a minimally invasive approach and exhibits superior safety and efficacy across multiple cancer types. Upon local light irradiation, PDT directly kills cancer cells via massive reactive oxygen species (ROS) generated by photosensitizers in an oxygen-dependent manner (Agostinis et al., 2011). Besides, PDT demonstrates great potential to polarize TAMs, offering a promising strategy to activate anticancer immunity (Li et al., 2024). Accumulating researches suggest that light-stimulated excessive ROS in tumor cells promotes immunogenic cell death (ICD), thereby activating TAMs. In this context, effective ICD induction in tumor cells necessitates intensive laser irradiation, a condition more feasible for surface tumor cells because light penetration decreases with depth due to progressive light absorption and scattering in tissue. As most of the photosensitizers lack tumor-targeting capability, their specific delivery into TAMs may serve as a powerful approach to directly modulate TAMs and eradicate tumors via PDT. Increasing reports have suggested that encapsulating photosensitizers into nanocarriers not only efficiently enhances their bioavailability but also improves their TAM targeting ability (Wang et al., 2019; Weissleder et al., 2014). Previously, we observed that positively charged dextran nanogels (nanoscale hydrogels) exhibited a superior TAM targeting efficacy (Deng et al., 2024), highlighting the potential of photosensitizer-loaded nanogels for light-induced TAM polarization.

To achieve superior anticancer PDT efficacy through TAM targeting and functional modulation, here we explore the positively charged diethylaminoethyl-dextran nano-photosensitizer (p-Dex PS) (Fig. S1, supplemental results and discussion in Supplementary Materials) for cancer PDT, focusing on TAM-mediated delivery, metabolic programming, TAM polarization, and anticancer immune effects. Chlorin e6 (Ce6) was selected as a model photosensitizer since it has been widely used in various PDT applications (Pham et al., 2021). As Ce6 nanocarrier control, the negatively charged carboxymethyl-dextran nano-photosensitizer (n-Dex PS) was also prepared (Fig. S2). Generally, the p-Dex PS and n-Dex PS demonstrate similar Ce6 loading efficiency (Table S1), comparable hydrodynamic size (Fig. S3), and stability (Fig. S4), but with different surface charges, which allow us to investigate the anticancer PDT efficacy through adjusting the TAM delivery efficiency. Considering nanocarriers would interact with both tumor cells and TAMs within the tumor microenvironment, the murine cancer cell line 4T1 and macrophage cell line RAW264.7 were used as model cells. As shown in Fig. S5A and S5D, the p-Dex PS delivered the highest Ce6, with similar uptake observed in tumor cells and macrophages, while n-Dex PS exhibited markedly reduced uptake by RAW264.7 cells, suggesting the positive dextran nanogels have a higher propensity to accumulate in macrophages (Deng et al., 2024). Upon light irradiation, p-Dex PS produced more intracellular ROS (Fig. S5E) and elicited much higher toxicity than that with n-Dex PS in 4T1 and RAW264.7 cells (Fig. S5F and S5G), while both of them exhibited minimal cytotoxicity in the absence of light at the tested Ce6 concentrations (Fig. S6). The superior phototoxicity of p-Dex PS was also validated through live/dead staining in 4T1 cells (Fig. S7), ascribing to the enhanced Ce6 delivery and the resultant ROS generation.

**Figure 1.**
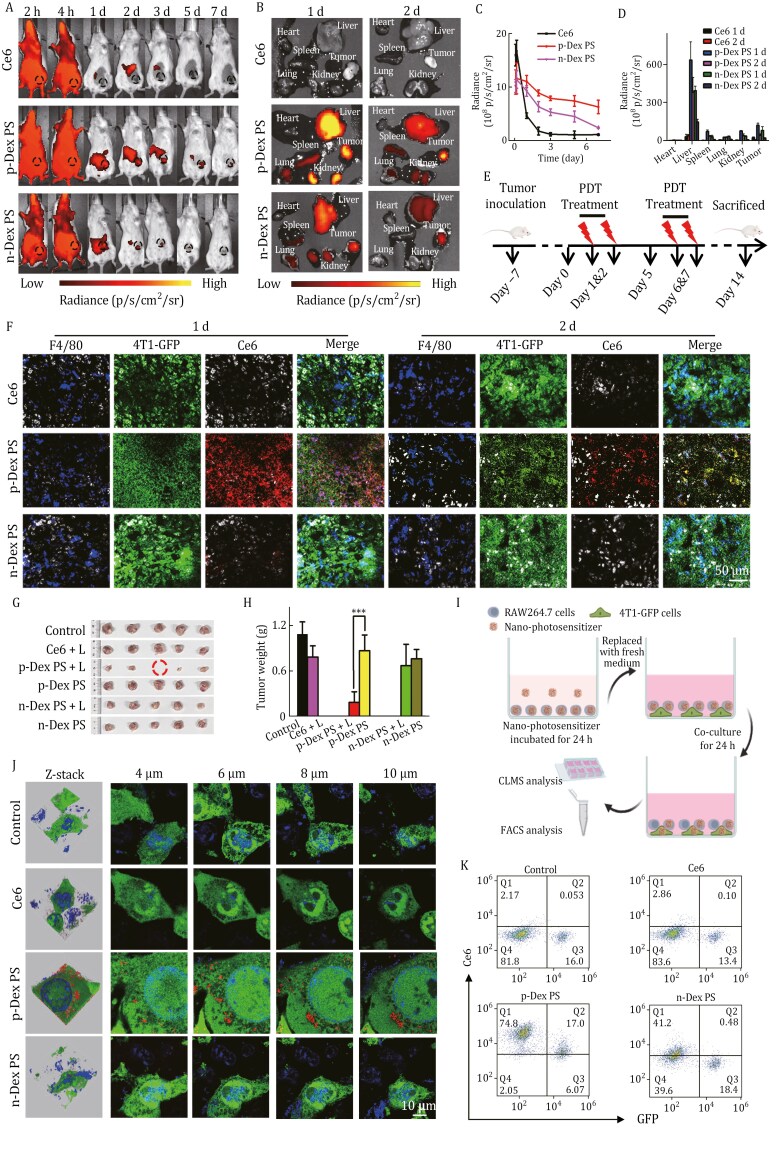
***In vivo* biodistribution, antitumor efficacy, and TAM-mediated delivery of p-Dex PS.** (A) Live animal imaging of p-Dex PS in 4T1-bearing mice within 7 days. The dashed lines represents the tumor site. Intravenous injection of equal doses of Ce6 and n-Dex PS was included as controls. (B) The biodistribution of p-Dex PS, n-Dex PS, and Ce6 in major organs and tumors at 1 and 2 days. (C) The fluorescent intensity obtained from the tumor area in Fig. 1A. (D) The fluorescent intensity of organs measured from *ex vivo* imaging in Fig. 1B. (E) Schematic illustration of the *in vivo* PDT procedures on 4T1 tumor-bearing mice. (F) The immunofluorescence images of tumor at 1 and 2 days after intravenous injection of nano-photosensitizers and Ce6. The blue, green, red, and gray signals represent the fluorescence of F4/80, 4T1-GFP, Ce6, and DAPI-stained nuclei. (G) Photographs of excised tumors. (H) Tumor weight. (I) Schematic illustration of the intercellular transport and redistribution of p-Dex PS based on the direct cell co-culture model. (J and K) The 3D reconstruction and multilayer confocal images (J) and the FACS quantification of mean Ce6 fluorescence intensity of 4T1-GFP cells (K).

Based on the intrinsic fluorescence of Ce6, we next determined the biodistribution of p-Dex PS in the immune-competent 4T1 tumor-bearing mice (Fig. 1A and 1B). Due to the rapid clearance of free Ce6, a negligible tumoral signal was found in the Ce6 group. While compared with n-Dex PS, intravenous administration of p-Dex PS displayed much higher fluorescence accumulation at tumor sites up to 7 days, suggesting an improved tumor-targeting of p-Dex PS (Fig. 1A and 1C), which was also verified by *ex vivo* imaging (Fig. 1B and 1D). Clear fluorescence in both liver and kidney for p-Dex PS and n-Dex PS after 1 day was observed, which could be explained by the altered Ce6 biodistribution that delivered by dextran nanogels (Cabral et al., 2024). Encouraged by this efficient tumoral delivery, the *in vivo* PDT effect was further evaluated in tumor-bearing mice treated by PBS (control), free Ce6, n-Dex PS and p-Dex PS with or without light irradiation (Fig. 1E). Compared to the control group, no significant inhibition of tumor growth was found in the treatment groups without light irradiation (Figs. 1G and S8). The p-Dex PS with light irradiation (p-Dex PS + L) displayed a stronger tumor inhibition rate than that of n-Dex PS + L group (81.73% vs. 32.11%) (Fig. 1H). The H&E and cell proliferating indicator Ki67 staining of the tumor slices, further confirmed the excellent anticancer PDT effects of p-Dex PS (Fig. S9). Additionally, the H&E staining of major organs did not exhibit any observable abnormality (Fig. S10A), which was correlated with the neglectable mouse weight variance during the treatment process (Fig. S8B). Meanwhile, serum biochemistry analyses of alkaline phosphatase (ALP), total protein (TP), and creatinine (CREA) revealed that p-Dex PS, either with or without light irradiation, had no significant effects on liver and kidney functions (Figs. S10B–D and S11), confirming the *in vivo* safety at the tested dose.

We further scrutinized the distribution of p-Dex PS within the tumor by examining Ce6 fluorescence in 4T1 cells constitutively expressing GFP (4T1-GFP) and TAMs highlighted by immunofluorescence staining with Cy3-labeled F4/80 antibody. At 1 day after intravenous injection, the Ce6 fluorescence of p-Dex PS colocalize well with that of TAMs, and its fluorescence intensity was significantly higher than that of n-Dex PS (Figs. 1F and S12). Although the fluorescence intensity of p-Dex PS group decreased somewhat 2 days after administration, consistent with *in vivo* imaging, greater colocalization of Ce6 with tumor cells was observed (Fig. S12). Based on these observations, we assumed that the p-Dex PS targeted to TAMs could be re-distributed into tumor cells. To test this, RAW264.7 cells loaded with nano-photosensitizer were co-cultured with 4T1 cells through both direct and indirect contact models, and the transfer of the nano-photosensitizer between cells was assessed using Ce6 fluorescence. As illustrated in Fig. 1I, the macrophage pretreated by p-Dex PS was directly co-cultured with 4T1-GFP cells, and clear Ce6 fluorescence transferred to 4T1-GFP cells was observed (19.55% ± 3.61%). While bare Ce6 fluorescence was found in 4T1-GFP cells in the cases of control, free Ce6 and n-Dex PS groups, with the Ce6 positive rates of 0.04% ± 0.02%, 0.08% ± 0.03%, and 0.63% ± 0.21%, respectively (Figs. 1J, 1K and S13). To further exclude the possible 4T1 cell’s internalization of the whole Raw264.7 cell loaded with Ce6, the macrophages with different pretreatments were cultured in the upper chamber of a transwell system with a pore size of 1 μm, and the lower chamber was seeded with 4T1 cells (Fig. S14A). After 24 h, the p-Dex PS group exhibited significantly higher Ce6 fluorescence in the upper medium (Fig. S14B), supporting an active excretion by the macrophage engulfing p-Dex PS. Moreover, this further led to a transfer of Ce6 from RAW264.7 to 4T1 cells as verified by FACS (Fig. S14C). Overall, the p-Dex PS engulfed by macrophages could be more efficient for the transport and redistribution of encapsulated Ce6 to 4T1 tumor cells than the cases of n-Dex PS and free Ce6, which potentially contributed to the superior phototoxicity observed *in vivo*.

**Figure 2.**
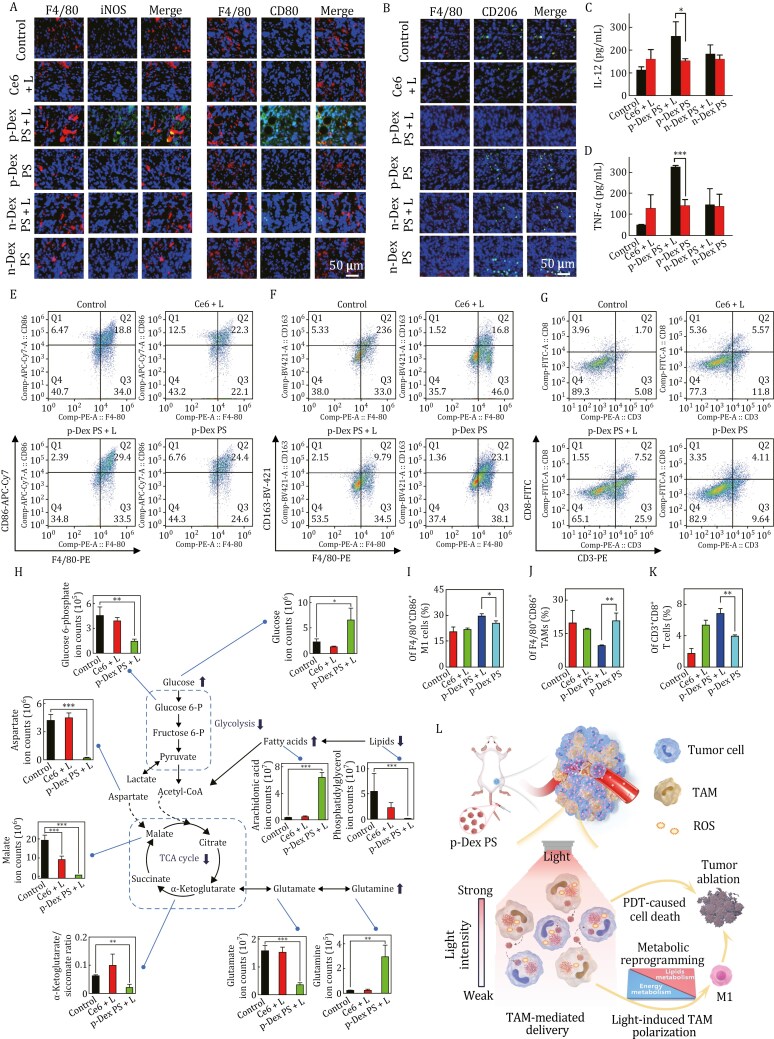
**The modulation of macrophage phenotypes, tumoral infiltration of immune cells, and the light-induced metabolic reprogramming after p-Dex PS treatment.** (A) The immunofluorescence images of iNOS and CD80 in tumor sections. The blue and red fluorescence represent the DAPI (nucleus) and F4/80 markers (macrophage), respectively. The green fluorescence represents CD80 or iNOS (M1 markers). (B) The immunofluorescence images of CD206 of tumor sections. The blue, green, and red signals represent the fluorescence of DAPI-stained nuclei, CD206 (M2 markers), and F4/80 markers. (C and D) ELISA assays of IL-12 (C) and TNF-α (D) in mice serum. (E) FACS analysis of F4/80^+^CD86^+^ M1-phenotype TAMs in tumor after control, Ce6 + L, p-Dex PS, and p-Dex PS + L treatments. (F) FACS analysis of F4/80^+^CD163^+^ M2-phenotype TAMs in tumor. (G) FACS analysis of CD3^+^CD8^+^ T cells in tumor. (H) The representative metabolic pathway induced by p-Dex PS in RAW 264.7 cells. (I–K) Quantitative data of intratumoral M1 (I), M2-phenotype TAMs infiltration (J) and CD8^+^ T cells infiltration (K) measured from FACS. (L) Schematic illustration of p-Dex PS for cancer immuno-PDT treatment. With the TAM-mediated delivery of p-Dex PS in tumor, the strong phototoxicity directly mediated cell killing while an attenuated light in deeper tumor tissue could induce TAM polarization.

To explore the mechanisms underlying the excellent PDT potency of p-Dex PS, we further assessed the TAMs phenotypes by staining excised tumors for iNOS and CD80 (M1 markers) and CD206 (an M2 marker). The p-Dex PS + L treatment clearly increased the proportion of M1 macrophages while decreasing the proportion of M2 macrophages within tumor (Fig. 2A and 2B). Compared with the control, p-Dex PS + L treatment also significantly induced a 2.3- and 6.5-fold increase for IL-12 and TNF-α in serum, respectively (Fig. 2C and 2D), which well confirmed the strongest TAM polarization effects of p-Dex PS upon light exposure. To validate the light-triggered immune activation of this p-Dex PS, intratumoral T cell infiltration and TAM populations were further analyzed. The viable cell population was selected for FACS gating as shown in Figs. S15 and S16. Compared with the control treatment, the p-Dex PS + L induced a modest decrease (~30%) of the whole TAM population, which could be resulted from the direct phototoxicity (Figs. S17 and S18). Within the gated TAM population, p-Dex PS + L led to a significant increase of M1-phenotype TAMs (F4/80^+^CD86^+^) and a decrease of M2-phenotype TAMs (F4/80^+^CD163^+^), when compared with other treatments (Fig. 2E, 2F, 2I, and 2J). Specifically, the intratumoral M1/M2 ratio in p-Dex PS + L group was the highest, corresponding to around 2.4- and 2.5-fold higher than that in the Ce6 + L and p-Dex PS groups, respectively (Fig. S19). Considering the reduction of the total number as well as polarization of TAMs play a crucial role in activation of intratumoral T cells to enhance the anticancer immunity (Cheng et al., 2022; Mantovani et al., 2022), the intratumoral CD8^+^ T cells infiltration was further assessed. As shown in Fig. 2G and 2K, 6.8% of CD8^+^ T cells infiltration in the p-Dex PS + L group was found in tumor, which was 4-, 1.3-, and 1.7-folds higher than that in the control, Ce6 + L and p-Dex PS groups, respectively. Besides, the ratio of tumoral CD4^+^ T cells in p-Dex PS + L group was also the highest among all treatments (Fig. S20). Taken together, these findings indicate light-triggered TAM polarization in the p-Dex PS + L group promotes antitumor immunity, thereby contributing to the excellent antitumor PDT efficacy.

To further probe the mechanism behind the light-mediated polarization of TAM when treated by p-Dex PS, metabolomics analysis was performed considering that metabolic variation could closely reflect the functional and phenotype changes in cells (Mehla and Singh, 2019). The light-induced polarization of RAW264.7 cells was firstly confirmed *in vitro* by assessing changes in M1 and M2 protein markers. Rather than focusing on the phototoxicity to eradicate tumor cells, a sublethal light dose (10 mW/cm^2^) was selected to perform the PDT experiments (Fig. S21). Due to limited light penetration *in vivo* caused by tissue scattering and absorption (Mallidi et al., 2016), the sublethal light dose used here simulates the attenuated light that deep-seated TAMs would experience, while minimizing macrophage activation linked to PDT-induced ICD. The p-Dex PS + L treatment resulted in a significantly higher CD80/CD206 ratio than free Ce6 and n-Dex PS, as determined by Western blot (Fig. S22A and S22B) and FACS (Fig. S22C). Meanwhile, p-Dex PS + L successfully increased the ratio of M1/M2 macrophage markers that were equivalent to lipopolysaccharide (LPS), a classical activator of macrophage polarization, indicating a great TAM reprogramming effect of p-Dex PS initiated by the light. Further metabolomics analysis showed that the light irradiation (10 mW/cm^2^) of p-Dex PS-treated cells could induce the most pronounced change in metabolite levels compared to n-Dex PS and free Ce6 groups either with or without light irradiation (Table S2). The p-Dex PS + L also caused the GSH consumption and a reduction in NAD/NADH ratio (Fig. S23), suggesting impaired electron transport chain and mitochondrial function. This was further supported by the dramatically reduced level of aspartate that indicates TCA cycling is blocked (Fig. 2H). Furthermore, p-Dex PS + L could inhibit central carbon metabolism, including glycolysis and glutaminolysis from the accumulation of glucose and glutamine, and the reduction of glutamate, glucose-6 phosphate. In addition, decreased amino acids and ATP level were also observed in p-Dex PS + L treated macrophages (Fig. S24), suggesting the down-regulated energy metabolism. And notably, the lipolysis was enhanced with p-Dex PS + L treatment, characterized by a low level of lipids (e.g., phosphatidylglycerol) and the accumulation of fatty acids (e.g., arachidonic acid) (Fig. 2H). Together with a lower ratio of α-ketoglutarate/succinate, higher levels of free fatty acids are indicative of M1 polarization (Fig. 2H) (Ecker et al., 2010; Liu et al., 2017; Menegaut et al., 2017). Collectively, with the macrophage targeted by p-Dex PS, light irradiation could induce metabolic reprogramming characterized by adaptive energy and lipid homeostasis, which was potentially associated with TAM polarization and thereby enhanced antitumor immunity alongside direct phototoxic effects against cancer cells (Fig. 2L).

To summarize, we realized a superior anticancer PDT based on p-Dex PS and unveiled the related immuno-photodynamic mechanism through focusing on the TAM-mediated delivery, polarization, and the metabolic rewiring. During anticancer PDT, following light penetration and attenuation in tumor tissue, while the phototoxicity kills tumor cells (e.g., inducing ICD) that are exposed to high light doses, our findings suggest sublethal light exposure may directly induce the polarization of TAMs, provided that the photosensitizer is effectively targeted. Taken together, this p-Dex PS served as not only an efficient delivery platform for TAM targeting but also offered a facile strategy to enable light-controlled macrophage polarization.

## Supplementary Material

pwaf064_Supplementary_Data

## Footnotes

The authors thank Ms. Weifang Cao from the Core Instrument Faculty of IBMS (CAMS & PUMC) for her assistance in the TEM experiments. This work was supported by the National Key R&D Program of China (2022YFA1205803), Haihe Laboratory of Cell Ecosystem Innovation Fund (22HHXBSS00040), Beijing Natural Science Foundation (7242277), Beijing Nova Program (Z201100006820110) of Beijing Municipal Science & Technology Commission, CAMS Innovation Fund for Medical Science (CIFMS 2024-I2M-TS-008, CIFMS 2021-I2M-1-006), and the Fundamental Research Funds for the Central Universities (3332021100).

## Notes

W.Z., L.W., H.X., and H.D. conceived the project idea, analyzed the data, and wrote the paper. H.D. and W.Z. prepared and characterized the nanogels. H.D., H.W., Y.Z., R.L., and W. H. performed the cell and animal experiments. L.W. and H.D. did the metabolomics studies. All authors read and edited the manuscript. All institutional and national guidelines for the care and use of laboratory animals were followed.

## References

[CIT0001] Agostinis P, Berg K, Cengel KA et al Photodynamic therapy of cancer: an update. CA Cancer J Clin 2011;61:250–281. 21617154 10.3322/caac.20114 PMC3209659

[CIT0002] Cabral H, Li JJ, Miyata K et al Controlling the biodistribution and clearance of nanomedicines. Nat Rev Bioeng 2024;2:214–232.

[CIT0003] Cheng HW, Fan XS, Ye EY et al Dual tumor microenvironment remodeling by glucose-contained radical copolymer for MRI-guided photoimmunotherapy. Adv Mater 2022;34:2107674. 10.1002/adma.202107674 34755922

[CIT0004] Deng H, Yang X, Wang HM et al Tailoring the surface charges of iron-crosslinked dextran nanogels towards improved tumor-associated macrophage targeting. Carbohydr Polym 2024;325:121585. 38008480 10.1016/j.carbpol.2023.121585

[CIT0005] Ecker J, Liebisch G, Englmaier M et al Induction of fatty acid synthesis is a key requirement for phagocytic differentiation of human monocytes. Proc Natl Acad Sci USA 2010;107:7817–7822. 20385828 10.1073/pnas.0912059107 PMC2867858

[CIT0006] Kzhyshkowska J, Shen JX, Larionova I. Targeting of TAMs: can we be more clever than cancer cells? Cell Mol Immunol 2024;21:1376–1409. 39516356 10.1038/s41423-024-01232-z PMC11607358

[CIT0007] Li XY, Gao JJ, Wu CC et al Precise modulation and use of reactive oxygen species for immunotherapy. Sci Adv 2024;10:eadl0479. 38748805 10.1126/sciadv.adl0479 PMC11095489

[CIT0008] Liu PS, Wang HP, Li XY et al alpha-ketoglutarate orchestrates macrophage activation through metabolic and epigenetic reprogramming. Nat Immunol 2017;18:985–994. 28714978 10.1038/ni.3796

[CIT0009] Mallidi S, Anbil S, Bulin AL et al Beyond the barriers of light penetration: strategies, perspectives and possibilities for photodynamic therapy. Theranostics 2016;6:2458–2487. 27877247 10.7150/thno.16183 PMC5118607

[CIT0010] Mantovani A, Allavena P, Marchesi F et al Macrophages as tools and targets in cancer therapy. Nat Rev Drug Discov 2022;21:799–820. 35974096 10.1038/s41573-022-00520-5 PMC9380983

[CIT0011] Mehla K, Singh PK. Metabolic regulation of macrophage polarization in cancer. Trends Cancer 2019;5:822–834. 31813459 10.1016/j.trecan.2019.10.007 PMC7187927

[CIT0012] Menegaut L, Thomas C, Lagrost L et al Fatty acid metabolism in macrophages: a target in cardio-metabolic diseases. Curr Opin Lipidol 2017;28:19–26. 27870652 10.1097/MOL.0000000000000370

[CIT0013] Pham TC, Nguyen VN, Choi Y et al Recent strategies to develop innovative photosensitizers for enhanced photodynamic therapy. Chem Rev 2021;121:13454–13619. 34582186 10.1021/acs.chemrev.1c00381

[CIT0014] Wang HR, Han X, Dong ZL et al Hyaluronidase with pH-responsive dextran modification as an adjuvant nanomedicine for enhanced photodynamic-immunotherapy of cancer. Adv Funct Mater 2019;29:1902440.

[CIT0015] Weissleder R, Nahrendorf M, Pittet MJ. Imaging macrophages with nanoparticles. Nat Mater 2014;13:125–138. 24452356 10.1038/nmat3780

