# Supplementary material for: Tumor-associated macrophage-mediated delivery of nano-photosensitizer enables light-induced metabolic programming for immuno-photodynamic therapy
Source: Protein Cell. 2025 Aug 20;16(12):1060–5. doi: 10.1093/procel/pwaf064 (PMC12742843; doi:10.1093/procel/pwaf064)
Supplement: pwaf064_Supplementary_Data [file pwaf064_supplementary_data.zip › pwaf064_suppl_Supplementary_Figure_1.docx]

Supplementary Materials

**Contents**

1. Supplemental results and discussion
2. Materials and methods

3. Table S1. The encapsulation and loading efficiency of Ce6 in p-Dex PS and n-Dex PS.

4.  Figure S1. Characterization of p-Dex PS.

5. Figure S2. Characterization of n-Dex PS.

6. Figure S3. The hydrodynamic size distribution, Zeta potential and PDI of p-Dex PS and n-Dex PS.

7. Figure S4. The stability of p-Dex PS and n-Dex PS.

8. Figure S5. Cellular uptake and PDT effects of p-Dex PS.

9. Figure S6. Dark cytotoxicity of Ce6, p-Dex PS and n-Dex PS.

10. Figure S7. Live-dead staining of 4T1 cells after PDT treatment with Ce6, p-Dex PS and n-Dex PS.

11. Figure S8. Tumor growth curves and body weight of tumor bearing mice after different treatments.

12. Figure S9. H&E and Ki67 staining of tumor slices after the treatments.

13. Figure S10. Biosafety of Ce6, p-Dex PS and n-Dex PS.

14. Figure S11. Serum biochemical parameters after treatment with Ce6 and p-Dex PS+L.

15. Figure S12. Immunofluorescence analysis of tumor frozen sections.

16.  Figure S13. Ce6 positive rate in 4T1 cells obtained from the direct coincubation of 4T1-GFP cells with RAW264.7 cells after different treatments.

17. Figure S14. The transportation efficiency of p-Dex PS from TAMs to tumor cells through the indirect contact model.

18. Figure S15. FACS gating strategy for M1- and M2-phenotype TAMs.

19. Figure S16. FACS gating strategy for CD8^+^ T cells.

20. Figure S17. FACS analysis of total TAMs in the tumor sites after different treatments.

21. Figure S18. Quantitative data of intratumoral TAMs infiltration after different treatments.

22. Figure S19. Quantitative data of the ratio of intratumoral M1/M2 TAMs after different treatments.

23. Figure S20. FACS gating strategy and analysis for CD4^+^ T cells after different treatments.

24. Figure S21. The cell viability of RAW264.7 cells treated with p-Dex PS at different light doses.

25. Figure S22. M1 and M2 markers expression determined by Western blot and FACS analysis after different treatments.

26. Figure S23. GSH and NAD/NADH ratio in RAW264.7 cells after treatment with control, Ce6 PDT and p-Dex PS PDT.

27. Figure S24. Variation of threonine, glycine and ATP in RAW 264.7 cells after the control, Ce6 PDT and p-Dex PS PDT treatments.

28. References.

29. Table S2. Variation of metobolites in RAW 264.7 cells after the control, Ce6, LPS, p-Dex PS and n-Dex PS treatments with or without light irradiation.

**Supplemental results and discussion**

**Preparation and characterization of p-Dex PS and n-Dex PS**

The p-Dex PS were prepared by the self-assembly of ferric ions, Ce6 photosensitizers and positively charged diethylaminoethyl-dextran (Fig. S1A) (Deng et al., 2024). As imaged by both transmission electron microscopy (TEM) and atomic force microscopy (AFM) (Fig. S1B-C), the p-Dex PS presented as amorphous nanogels with a hydrodynamic size of 160 nm and a PDI value of 0.17 (Fig. S3). In the meantime, the p-Dex PS displayed a good stability with almost unchanged size through 14 days observation of the hydrodynamic size (Fig. S4). To verify the construction of p-Dex PS, the UV-vis and fluorescence spectra were measured as shown in Fig. S1D-E. p-Dex PS showed broaden absorption bands at Soret band, red-shifted of about 30 nm at Q-bands and quenched fluorescence, which may be due to the noncovalent interactions of aggregated Ce6 in the nanogels (Zhu et al., 2022). To probe the noncovalent interactions involved in the formation of p-Dex PS, we attempted to analyze the ^1^H NMR spectra of the lyophilized nanogels and their components in deuterium solvents. The proton signals from Ce6 could be observed in p-Dex PS by ^1^H NMR (Fig. S1H). We found distortion signals at H_a_-_d_ in p-Dex PS compared with those of the parent Ce6, suggesting the coordination of carboxy groups on Ce6 with ferric irons on p-Dex PS. In addition, the chemical shift of H_e_ in p-Dex PS showed a significant up-field shift when compared to that in diethylaminoethyl-dextran, indicating ion pair and hydrogen-bond interaction between protons on diethylaminoethyl-dextran and carboxyl anions on Ce6. The successful assembly of irons, Ce6 and diethylaminoethyl-dextran led to a Ce6 loading efficiency of 5.61% (Table S1). Considering the ROS generation ability plays a pivotal role in determining PDT efficacy, the singlet oxygen (^1^O_2_) generation of p-Dex PS was evaluated by using the SOSG assay. Compared to free Ce6 under laser irradiation, ROS generation of p-Dex PS was apparently weakened when dispersed in aqueous solution (Fig. S1G), possibly due to Ce6 aggregation in the nanogels. This quenched ROS generation was expected to recover following the uptake of p-Dex PS and Ce6 release in cells. The zeta potential of p-Dex PS was ~40 mV (Fig. S3B).

As the nano-photosensitizer control, the negatively charged dextran nanogels that are composed of carboxymethyl-dextran and Ce6 (designated as n-Dex PS) (Fig. S2) were also prepared following the same protocol for p-Dex PS. As shown in Fig. S2D-G, the n-Dex PS demonstrated a similar absorption spectra shift, and quench of both fluorescence and singlet oxygen generation, which suggested similar Ce6 aggregation within both n-Dex PS and p-Dex PS. However, we found it was difficult to analyze the interactions between Ce6 and carboxymethyl-dextran in the formation of n-Dex PS solely using ^1^H NMR spectra, as the proton signals from Ce6 could not be found in n-Dex PS, probably ascribing to the poor solubility of lyophilized n-Dex PS (Fig. S2H). Nevertheless, the sustained release of Ce6 from n-Dex PS but with different speed could also be spotted (Fig. S1F and S2F), which might be resulted from the varied interaction strength of Ce6 within these two nanogels that carry differently charged side groups.

**Materials and methods**

**Materials and instruments**

Diethylaminoethyl-dextran (40 kDa, #80881), chlorin e6 (Ce6) and red blood cell lysis buffer were purchased from Sigma Aldrich (St. Louis, MO, USA). Carboxymethyl-dextran (40 kDa, #C866294) was from Macklin Reagent (Shanghai, China). Calcein AM, propidium iodide (PI), Cell Counting Kit-8 (CCK-8), 2,7-dichlorodihydrofluorescein diacetate (DCFH-DA) and Singlet Oxygen Sensor Green (SOSG) were from Beyotime Biotechnology (Shanghai, China). Dialysis membrane (MWCO 3.5 kDa) was from Thermo Fisher Scientific (Waltham, MA, USA). FITC-labeled anti-CD80 antibody (clone 16-10A1) and anti-CD206 antibody (clone C068C2), Percp-Cy5.5-labeled anti-CD45 antibody (clone 30-F11), PE-labeled anti-CD3 antibody (clone 17A2), FITC-labeled anti-CD8 antibody (clone 53-6.7), APC-Cy7-labeled anti-CD4 antibody (clone RM4-5), APC-labeled anti-CD11b antibody (clone M1/70), PE-labeled anti-F4/80 antibody (clone BM8), APC-Cy7-labeled CD86 antibody (clone GL-1), BV421-labeled CD163 antibody (clone S15049I) and Zombie Aqua (#423101) were purchased from BioLegend (San Diego, CA, USA). Anti-CD80 antibody (sc-376012), anti-iNOS antibody (sc-7271) and the secondary antibodies were purchased from Santa Cruz Biotechnology (Dalla, TX, USA). Anti-CD206 antibody (ab64693) was from Abcam (Cambridge, UK). Anti-F4/80 antibody (clone BM8) was obtained from eBioscience (San Diego, CA, USA). Collagenase Type 1 (#17100-017) was purchased from GIBCO (Waltham, MA, USA). All other agents used in the experiments were analytical grade from Beijing Chemical Plant (Beijing, China).

**Synthesis of dextran nano-photosensitizers**

The synthesis of dextran nano-photosensitizers was according to our recently published article (Deng et al., 2024). Briefly, for p-Dex PS, 800 μL of diethylaminoethyl-dextran (2.5 mg/mL) was added into the mixed solution with 40 μL of Ce6 (15 mM) and 160 μL of ferric chloride (1.6 mM) at 90 °C. For n-Dex PS, 800 μL of carboxymethyl-dextran (2.5 mg/mL) was added into the mixed solution with 22 μL of Ce6 (15 mM) and 178 μL of ferric chloride (8 mM) at 90 °C. The above mixture was further incubated at 90 °C for 30 min. Then the solution was cooled on ice for 10 min and purified by dialysis in pure water (MWCO=3.5 kD). The concentration of Ce6 in these nano-photosensitizers was then calculated according to the standard curve of Ce6 in 10 mM NaOH. The encapsulation and loading efficiency of Ce6 were calculated by the following equations:

$$\text{Encapsulation efficiency of Ce6}= \frac{\text{Ce6 retained in nanogel}\text{s}}{\text{Ce6 added in nanogel}\text{s}} \text{100\%}$$

$$\text{Loading efficiency of Ce6}= \frac{\text{Ce6 retained in nanogels}}{\text{Total weight of nanogels}} \text{100\%}$$

**Characterization of the nano-photosensitizers**

Transmission electron microscopy (TEM) images of the nanogels were recorded on a JEM-1400Plus TEM (JEOL Ltd., Tokyo, Japan). Atomic force microscopy (AFM) images were measured with a Dimension Icon AFM (Bruker, Billerica, Massachusetts, USA). UV-vis absorption and fluorescence spectra were determined with the UH-5300 and F-7000 spectrophotometers, respectively (Hitachi Ltd., Tokyo, Japan). Hydrodynamic size distribution and Zeta potential were determined by a Zetasize Nano ZS90 (Malvern Ltd., Worcestershire, UK). ^1^H NMR spectra were acquired with a Bruke Avance Ⅲ 400 MHz NMR spectrometer (Bruke Ltd., Rheinstetten, Germany). Lyophilized powders of p-Dex PS and their components were dissolved in DMSO-d_6_ (δ = 2.50 ppm) for ^1^H NMR analysis. Lyophilized powders of n-Dex PS and their components were dissolved in D_2_O (δ = 4.79 ppm) for ^1^H NMR analysis. Flow cytometry (FACS) analysis was determined with a BD Accuri C6 (BD Biosciences, Franklin Lakes, NJ, USA). In vitro fluorescence images were recorded with an EVOS fluorescent microscope (Life Technologies, Carlsbad, CA, USA) and a Leica SP8 confocal microscope (Leica Microsystems, Wetzlar, Germany). In vivo fluorescence images were obtained by a Xenogen IVIS spectrum system (Xenogen, Alameda, CA, USA).

**Drug release assay**

To determine the Ce6 release, 1 mL of nanogels with 1 mL of FBS was placed into the dialysis bag, and then immersed in 80 mL of PBS at 37 °C with magnetic stirring. At predetermined time points, 1 mL of PBS was collected, and equal volume of fresh PBS was replenished. The concentration of Ce6 was calculated according to the standard curve of Ce6.

**Singlet oxygen generation experiments**

The production of ^1^O_2_ was detected by SOSG as a detection probe. p-Dex PS, n-Dex PS and Ce6 (the Ce6 concentration of 500 nM) were dispersed in SOSG PBS solution (20 μM). Only SOSG in PBS was set as the control. Then the mixture was irradiated with a 650 nm laser (60 mW/cm^2^) at the fixed time interval (0, 0.25, 1, 2, 3, 4, 5 and 6 min). The production of ^1^O_2_ was studied by measurement of the fluorescence intensity at 525 nm.

**Intracellular ROS detection**

Intracellular ROS detection was determined by DCFH-DA probes with RAW264.7 and 4T1 cells (Cell Resource Center of Chinese Academy of Medical Sciences & Peking Union Medical College, Beijing, China). Cells were seeded in 96-well plates until subconfluence. The cells were treated with p-Dex PS, n-Dex PS and Ce6 (500 nM Ce6) in the dark for 24 h, and then exposed to 10 µM DCFH-DA for 30 min. After that, the cells were irradiated under a 650 nm laser (60 mW/cm^2^, 30 s). The generation of ROS was measured by fluorescence imaging under the GFP channel.

**Cellular uptake of nano-photosensitizers**

4T1 and RAW264.7 cells were seeded in 96-well plates and treated with p-Dex PS, n-Dex PS and Ce6 (500 nM Ce6) for 24 h. After being washed with PBS, the cells were incubated with Hoechst for 2 min, followed by observation under DAPI and Cy5 channels, using an EVOS fluorescent microscope. And the cellular uptake of Ce6 was also evaluated in Cy5 channel by FACS analysis.

**Cell viability and live/dead staining**

4T1 and RAW264.7 cells were treated with various concentrations of Ce6 or nanogels (p-Dex PS or n-Dex PS) for 24 h. After that, the cells were placed under a 650 nm laser (60 mW/cm^2^) or the dark for 60 s. After overnight incubation, the cell viability was analyzed by CCK-8 assay kit. For live/dead staining, 4T1 cells were incubated with p-Dex PS, n-Dex PS and Ce6 containing 0.25-1 μM Ce6 for 24 h. Next, the cells were treated by a 650 nm laser (60 mW/cm^2^, 60 s). After overnight incubation, the cells were double stained with Calcein-AM (2 µM) and propidium iodide (PI) (5 µM) for 6 min. The fluorescence images were recorded in GFP and RFP channels, respectively.

**In vitro redistribution of nano-photosensitizers by co-culture of RAW264.7 cells with 4T1 cells**

The intercellular transport and redistribution of nano-photosensitizers were observed by direct co-culture of treated RAW264.7 cells with 4T1-GFP cells. 4T1-GFP cells were obtained from the Institute of Laboratory Animal Sciences, the Chinese Academy of Medical Sciences & Peking Union Medical College. RAW264.7 cells were firstly seeded in the 12-well plates and treated with p-Dex PS, n-Dex PS, Ce6 (500 nM Ce6) and drug-free medium for 24 h, and then rinsed with PBS. After that, the RAW264.7 cells were collected, co-cultured with 4T1-GFP cells in the 24-well plates for 24 h. Subsequently, both cells were collected and analyzed by FACS in GFP and Cy5 channel. For Confocal Laser Scanning Microscope (CLSM) observation, the RAW264.7 cells were harvested and co-cultured with 4T1-GFP cells in the 8-well chamber slides for 24 h. Then the co-cultured cells were observed by CLSM (Leica SP8 confocal microscope).

**Transwell assay**

Transwell plates with a pore size of 1 μm were used to investigate the intercellular transport and redistribution of nano-photosensitizers. This pore size would hinder the direct contact of macrophages with 4T1 cells, while allowing the translocation of secreted vesicles or nano-photosensitizers. The RAW264.7 cells were seeded in the upper chamber of the transwell and treated with p-Dex PS, n-Dex PS, Ce6 (500 nM Ce6) and drug-free medium for 24 h. After the drug-containing medium was placed by fresh drug-free medium, the RAW264.7 cells in the upper chamber were co-cultured with 4T1 cells in the bottom plate of the transwell for 24 h. Then the medium in the upper chamber was collected and its fluorescence intensity at 670 nm was measured with a microplate reader (Thermo Fisher Scientific). And the 4T1 cells in the bottom plate were harvested and analyzed by FACS analysis in Cy5 channel.

**Biodistribution and biochemical analysis**

Female BALB/c mice (Beijing Vital River Laboratory Animal Technology Co., Ltd, Beijing, China) bearing 4T1 tumors were used for in vivo investigation. All animal procedures adhered to the National Research Council's Guide for the Care and Use of Laboratory Animals and were approved by the Institutional Animal Care and Use Committee of Chinese Academy of Medical Sciences & Peking Union Medical College (ACUC-A02-2021-005). The 4T1 tumor bearing mice were intravenously injected with p-Dex PS, n-Dex PS and Ce6 (5 mg/Kg Ce6). For in vivo biodistribution, the fluorescence images of 4T1 tumor bearing mice were acquired within 7 days using a Xenogen IVIS spectrum system. The images were obtained under 640 nm excitation and 680 nm emission. The major organs and tumors of mice were excised for ex vivo fluorescence imaging at 1- and 2-days post injection. For biochemical analysis, serum samples were collected from mice at 2-days post injection of PBS, Ce6, p-Dex PS and n-Dex PS with or without laser irradiation. A 650 nm laser (500 mW/cm^2^, 5 min) was used for irradiation at 1-day after the injection. Alkaline phosphatase (ALP), total protein (TP) and creatinine (CREA) were measured as indicators of hepatic and renal functions. Tumors from 4T1-GFP tumor-bearing mice were excised for frozen section at 1- and 2-days post injection. After being stained with Cy3-labeled F4/80 antibody, the slides were treated with mounting medium containing DAPI. The CLSM images were obtained using DAPI, GFP, RFP and Cy5 channels.

**In vivo antitumor activity**

Female 4T1 tumor-bearing mice were randomly divided into 6 groups: (1) PBS; (2) Ce6 plus laser irradiation (Ce6+L); (3) p-Dex PS plus laser irradiation (p-Dex PS+L); (4) p-Dex PS; (5) n-Dex PS plus laser irradiation (n-Dex PS+L); (6) n-Dex PS. Different formulations with a Ce6 dose of 5 mg/Kg were intravenously injected at 1 day prior to the first irradiation and 5 days post the first injection, respectively. Then the 650 nm laser (500 mW/cm^2^, 5 min) was used for irradiation at 1- and 2-days post injection. Body weight and tumor volume of each mouse were recorded every 2 days. At the end of treatment, the major organs and tumors were harvested for H&E staining. Tumors were weighed and collected for ki67 staining. For immunofluorescence staining, the tumor sections were stained with F4/80, CD80, iNOS and CD206 antibodies. And the serum sample of each group was also collected for measurement of IL-12 and TNF-α by ELISA (Neobioscience Technology, Shenzhen, China).

**Immune cells infiltration in tumor using FACS analysis**

The 4T1 tumor-bearing mice were processed with PBS, Ce6 plus laser, p-Dex PS and n-Dex PS plus laser, respectively, with the Ce6 concentration of 5 mg/Kg. At 1 and 2 days after the injection, the mice were treated with the antitumor irradiation dose described above. At 6 days post injection, the tumor tissues were harvested and FACS analysis was used to analyze the tumoral immune cells infiltration. The tumor tissues were digested with collagenase (1 mg/mL) to collect the cells at 37 °C for 1 h. Then Ack lysis buffer was added in the cells for lysis of erythrocytes at 4 °C for 5 min. After being rinsed with PBS, the obtained single cell suspension was stained with Zombie Aqua viability kit at room temperature for 20 min to select viable cells for further experiments, and subsequently split into two. Then one cell sample was stained in the fluorescence-labeled antibodies of cytotoxic CD8^+^ T lymphocytes (CD45^+^CD3^+^CD8^+^) and CD4^+^ T lymphocytes (CD45^+^CD3^+^CD4^+^) at 4 °C for 30 min. The other cell sample was stained in a series of fluorescence-labeled antibodies: M1-phenotype TAMs (CD45^+^CD11b^+^F4/80^+^CD86^+^) and M2-phenotype TAMs (CD45^+^CD11b^+^F4/80^+^CD163^+^) at 4 °C for 30 min. The immune cell infiltration of the two samples were measured using FACS.

**In vitro evaluation of macrophage types**

The light dose-dependent toxicity of RAW264.7 cells after being treated with the dextran nanogels were determined using CCK-8 assay in the dark or a 650 nm laser with the density of 5, 10, 15 and 20 mW/cm^2^ for 60 s. A sublethal light dose (10 mw/cm^2^) was then selected to evaluate the effects of the dextran nanogels on macrophage polarization. The RAW264.7 cells were treated with p-Dex PS, n-Dex PS, Ce6 (500 nM Ce6), lipopolysaccharide (LPS, 1 µg/mL) and drug-free medium for 24 h, and further exposed to the dark or the laser (10 mW/cm^2^). For FACS analysis, the treated cells were harvested and stained with FITC-labeled anti-CD80 antibody or anti-CD206 antibody at concentrations of 2 µg/mL in cold PBS for 30 min. The stained cells were analyzed by FACS in FL1 channel. For Western blot, above stimulated cells were harvested and proteins of cell lysates were prepared. Then the proteins of different groups were loaded on an 8% SDS-PAGE and transferred to PVDF membranes. The PVDF membranes were incubated with primary antibodies (anti-CD80 antibody and anti-CD206 antibody, 1/500 dilution) and then incubated with the secondary antibodies (1/5000 dilution). Finally, the signals were obtained by a Tanon 4800Muti Gel Imaging Analysis System (Shanghai, China).

**Metabolomics analysis**

RAW264.7 cells were treated with p-Dex PS, n-Dex PS, Ce6 (500 nM Ce6), lipopolysaccharide (LPS, 1 µg/mL) and drug-free medium for 24 h. And after being rinsed with PBS, the cells were exposed to the dark or a 650 nm laser with the density of 10 mW/cm^2^ for 60 s. Then the treated RAW264.7 cells were quenched with extraction buffer (methanol 80%) and exposed on ice throughout all metabolite extraction process. Cells were collected in Eppendorf tubes and centrifuged at 14700 rpm for 30 min at 4 °C. The supernatant was stored at -80°C until LC-MS analysis. LC separation was achieved using a Vanquish UHPLC system (Thermo Fisher Scientific) and an Xbridge BEH Amide column (150 × 2 mm, 2.5 µm particle size; Waters, Milford, MA, USA). Solvent A is 95:5 water: acetonitrile with 20 mM ammonium acetate and 20 mM ammonium hydroxide at pH 9.4, and solvent B is acetonitrile. The gradient is 0 min, 90% B; 2 min, 90% B; 3 min, 75% B; 7 min, 75% B; 8 min, 70% B; 9 min, 70% B; 10 min, 50% B; 12 min, 50% B; 13 min, 25% B; 14 min, 25% B; 16 min, 0% B; 21 min, 0% B; 23 min, 90% B; 25 min, 90% B. Total running time is 25 min at a flow rate of 150 µl/min. For all experiments, 8 µl of extract was injected with column temperature set to 25°C. The Orbitrap Exploris^TM^ 480 mass spectrometer (Thermo Fisher Scientific) was operated in both negative and positive mode scanning m/z 70-1000 with a resolution (at m/z 200) of 120,000. MS parameters are as follows: sheath gas flow rate, 28 (arbitrary units); aux gas flow rate, 10 (arbitrary units); sweep gas flow rate, 1 (arbitrary units); spray voltage, 3.3 kV; capillary temperature, 320°C.

**Statistical analysis**

Two-tailed Student's t-test was used for statistical analysis. A p-value less than 0.05 was considered statistically significant (*p < 0.05, **p < 0.01 and ***p < 0.001). All the data were presented as Mean ± SD.

Table S1. The encapsulation and loading efficiency of Ce6 in p-Dex PS and n-Dex PS.

| Nano photosensitizers | The encapsulation  efficiency of Ce6 | Ce6 loading efficiency (wt.%) |
| --- | --- | --- |
| p-Dex PS | 30.15±2.11% | 5.61±0.38% |
| n-Dex PS | 54.43±1.77% | 5.54±0.15% |


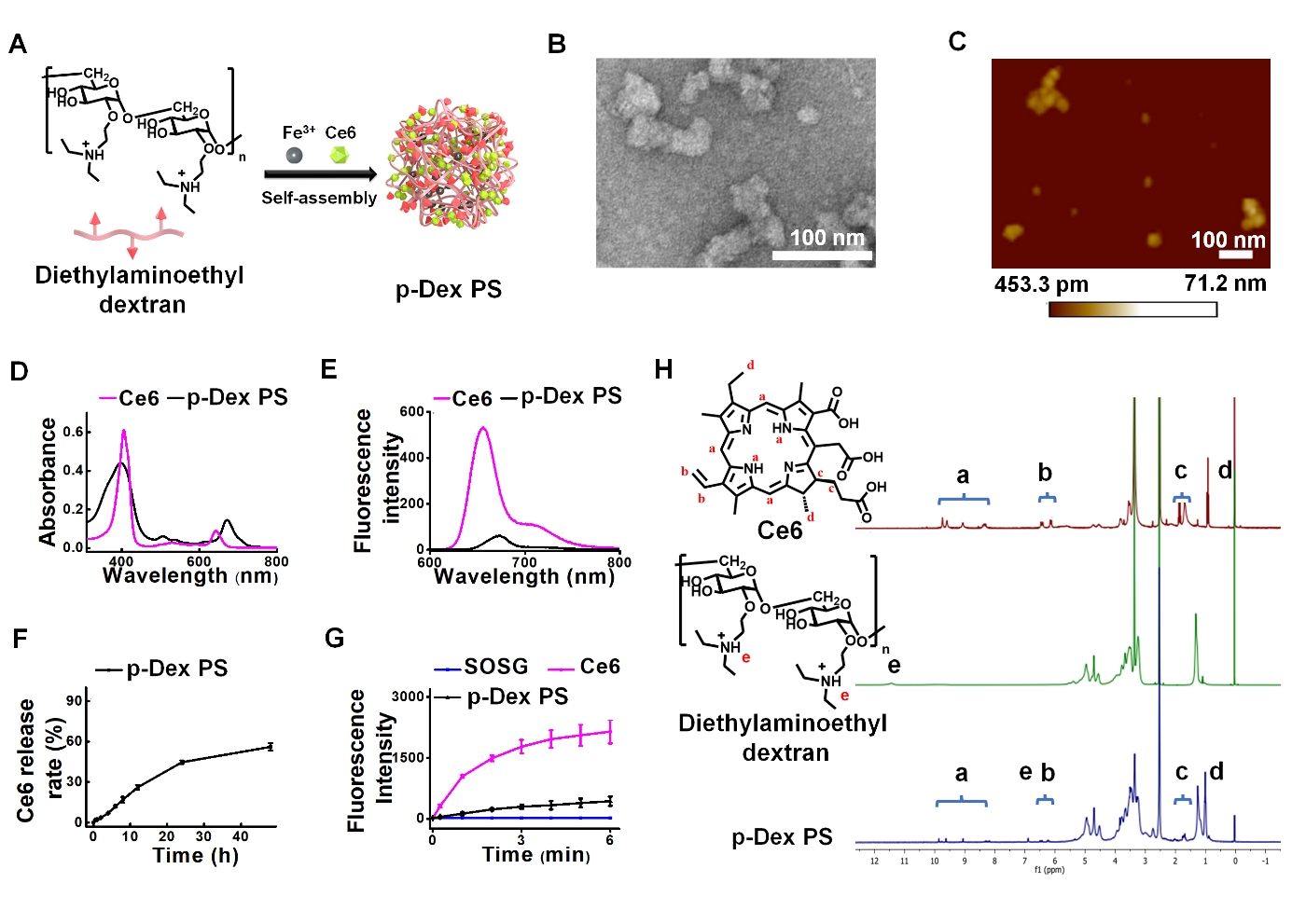


Figure S1. (A) Schematic illustration of the construction of p-Dex PS. (B-C) TEM and AFM images of p-Dex PS. (D) UV-vis absorption spectra and (E) fluorescence spectra of p-Dex PS. (F) Ce6 release curve of p-Dex PS in PBS (pH 7.4) in the presence of FBS. (G) ^1^O_2_ generation ability of p-Dex PS compared with Ce6 in PBS solution. SOSG only was set as control. (H) ^1^H NMR spectra of Ce6, diethylaminoethyl dextran and p-Dex PS.


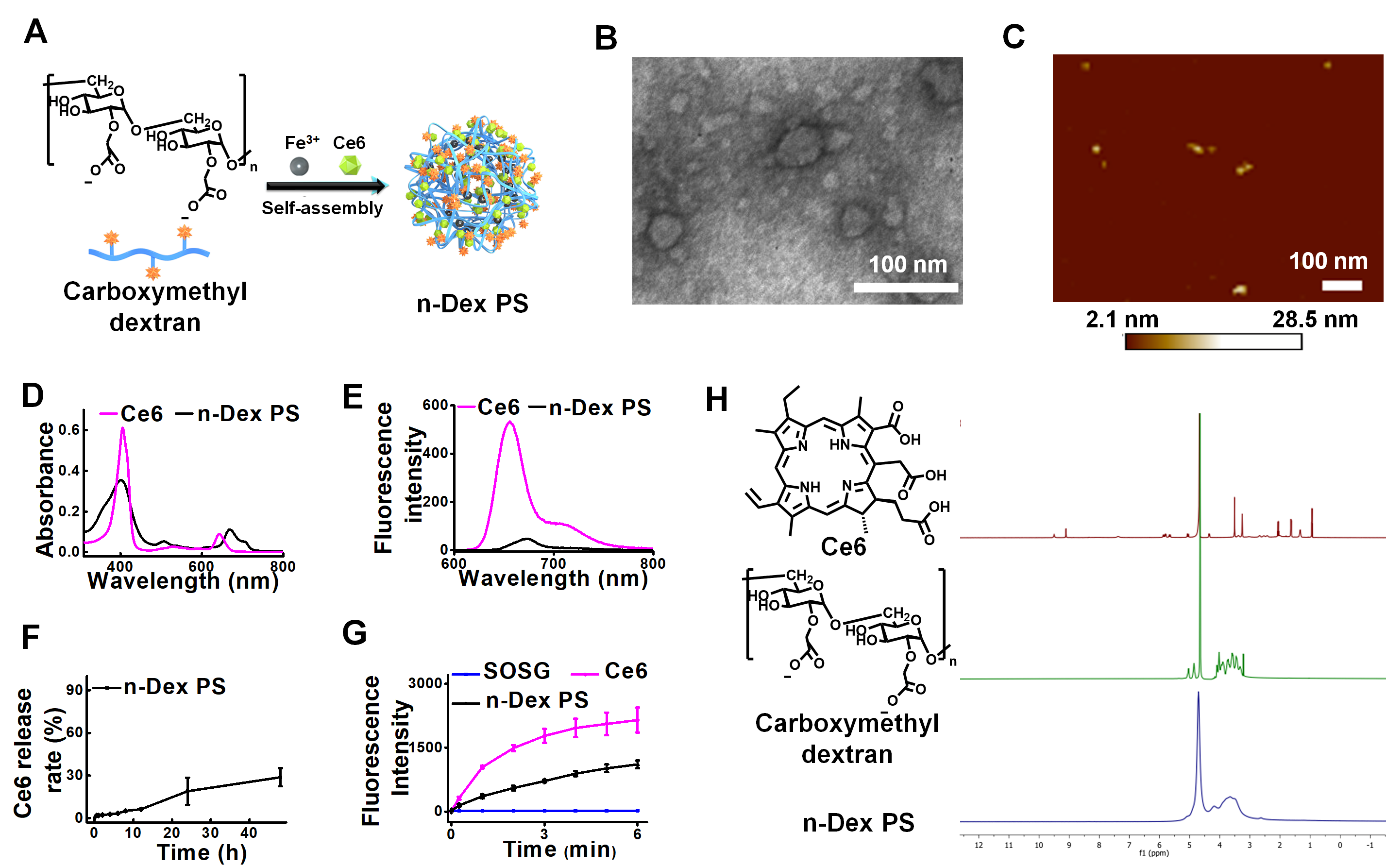


**Figure S2.** (A) Schematic illustration of the construction of n-Dex PS . (B-C) TEM and AFM images of n-Dex PS. (D) UV-vis absorption spectra and (E) fluorescence spectra of n-Dex PS. (F) Ce6 release curve of n-Dex PS in PBS (pH 7.4) with the presence of FBS. (G) ^1^O_2_ generation ability of n-Dex PS compared with Ce6 in PBS solution. SOSG only was set as control. (H) ^1^H NMR spectra of Ce6, carboxymethyl-dextran and n-Dex PS.


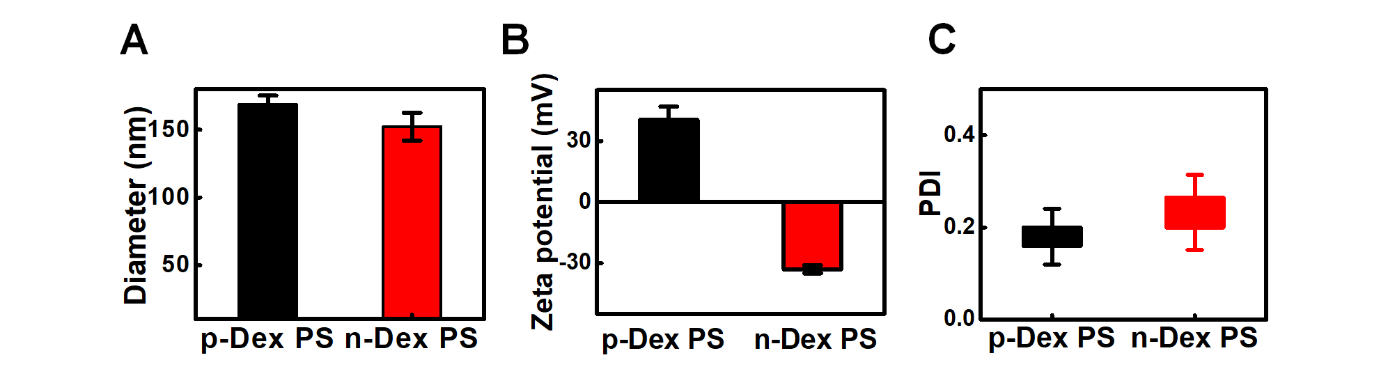
 Figure S3. (A) The hydrodynamic size distribution, (B) Zeta potential and (C) PDI of p-Dex PS and n-Dex PS.


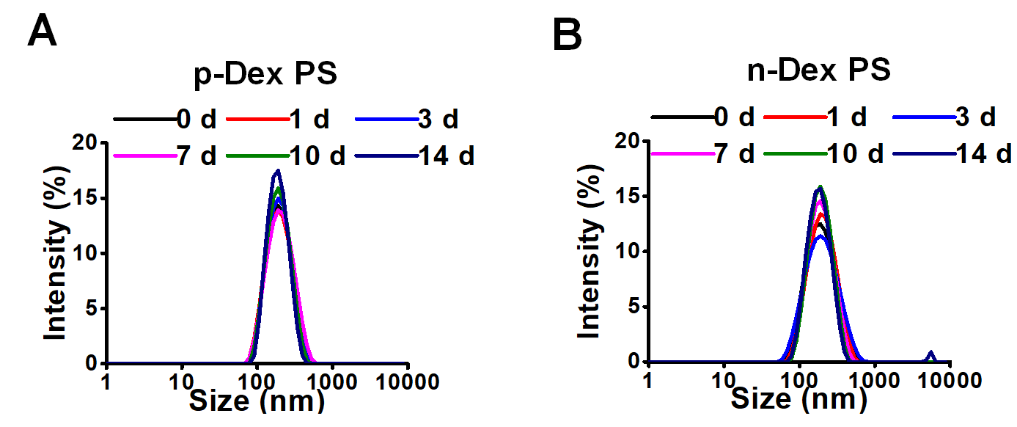


Figure S4. The hydrodynamic size distribution of p-Dex PS and n-Dex PS stored at 4°C for 14 days.


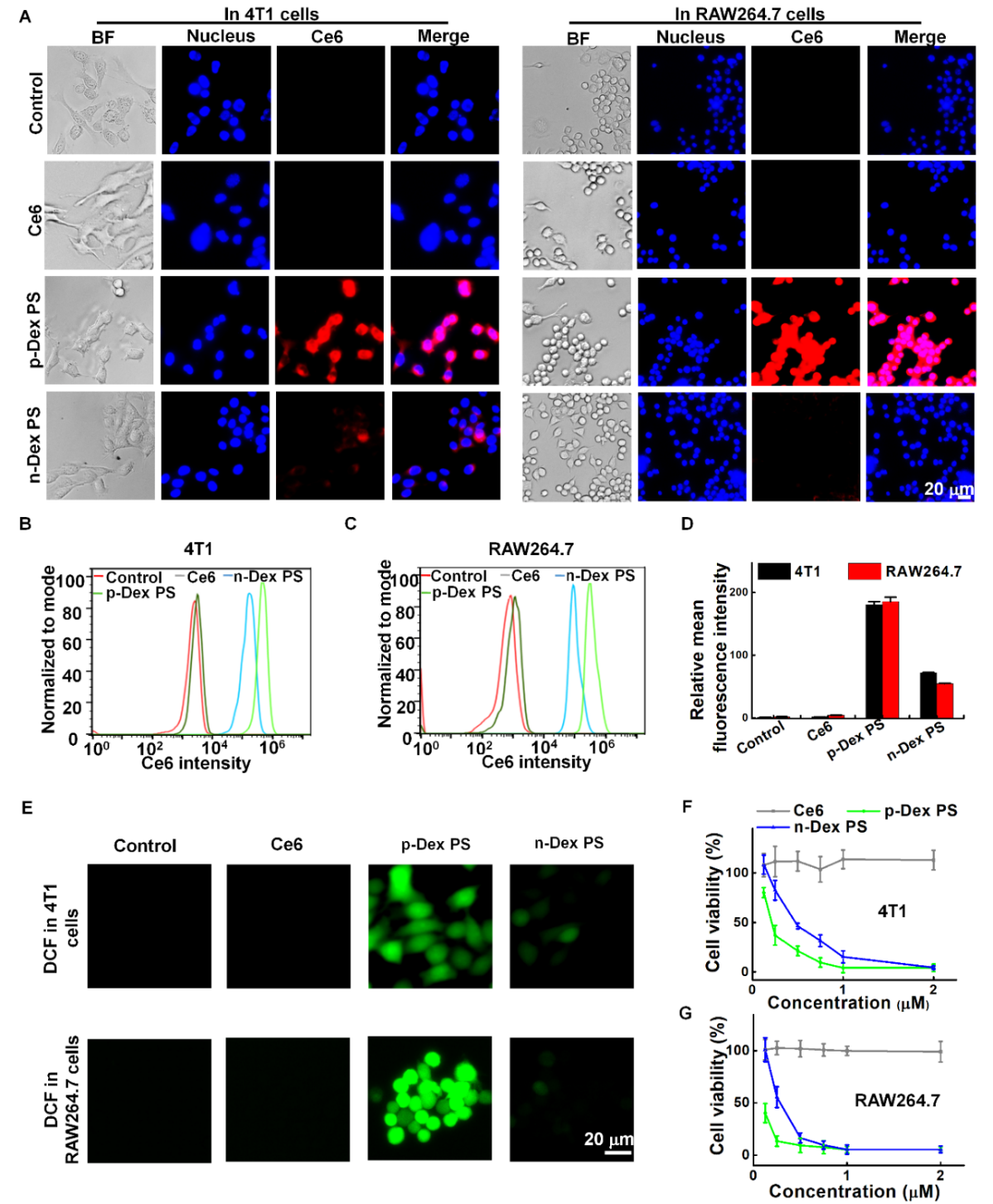


**Figure S5.** (A) Fluorescence images of 4T1 and RAW264.7 cells incubated with p-Dex PS for 24 h. Free Ce6 and n-Dex PS were included as controls. (B-C) Representative FACS graphs of Ce6 uptake in 4T1 and RAW264.7 cells after treatment with different groups. (D) Relative mean fluorescence intensity of Ce6 in cells determined from FACS. (E) Fluorescence images of 4T1 and RAW264.7 cells stained with DCFH-DA. (F-G) Phototoxicity of different nano-photosensitizer and Ce6 to 4T1 and RAW264.7 cells.


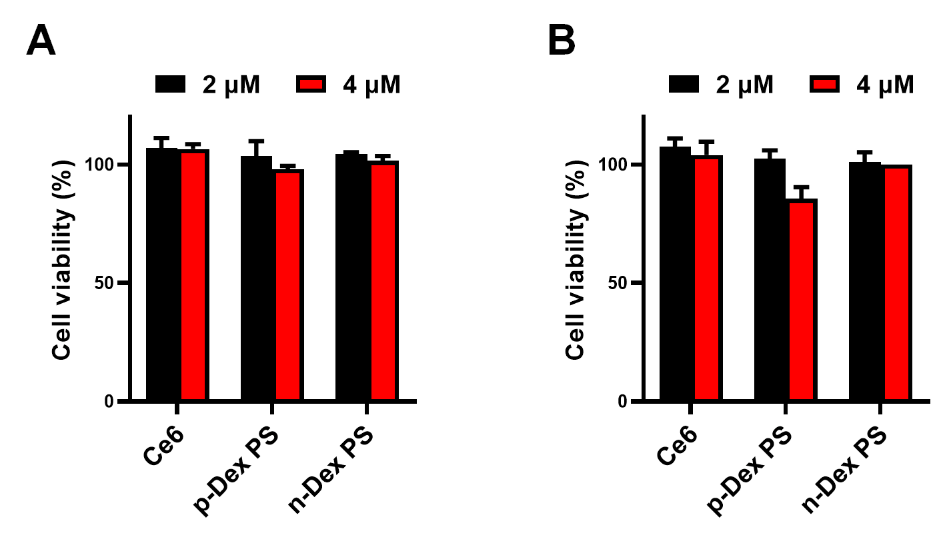


**Figure S6.** Dark cytotoxicity of Ce6, p-Dex PS and n-Dex PS to RAW264.7 (A) and 4T1 cells (B), with Ce6 concentration of 2 µM and 4 µM.


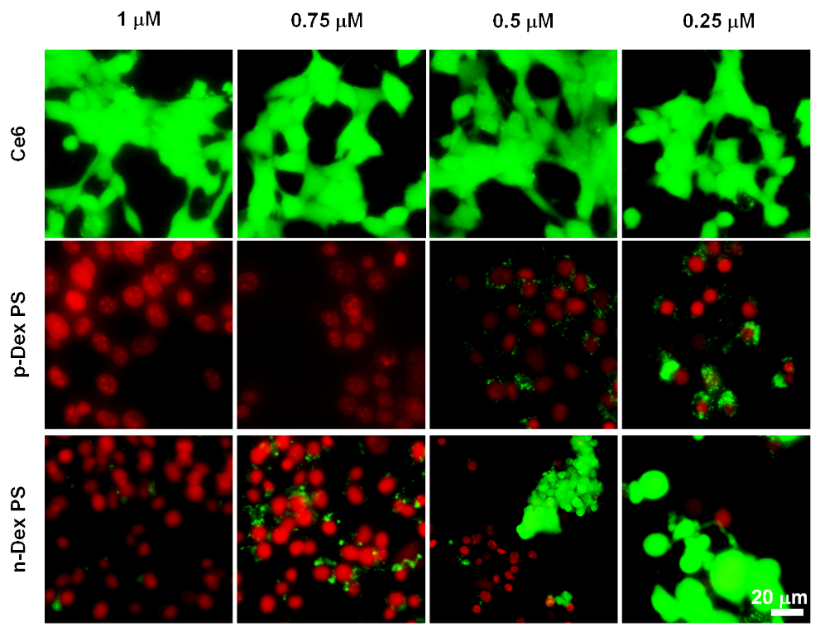


**Figure S7.** Live-dead staining of 4T1 cells after PDT treatment with Ce6, p-Dex Ps and n-Dex PS of varied Ce6 concentration. The green and red fluorescence represent live (Calcein-AM) and dead (PI) staining of cells, respectively.


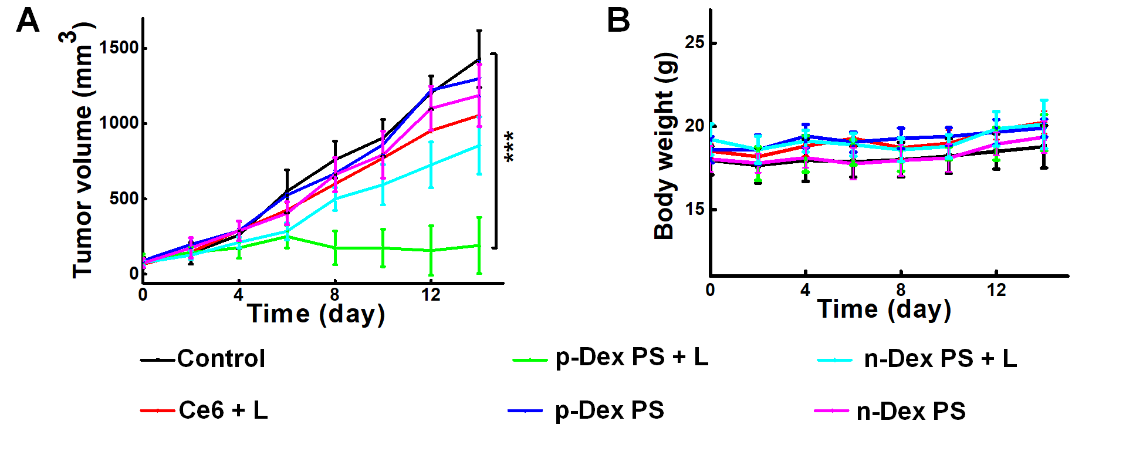


**Figure S8.** (A) Tumor growth curves after different treatments. (B) Body weight of 4T1 tumor bearing mice.


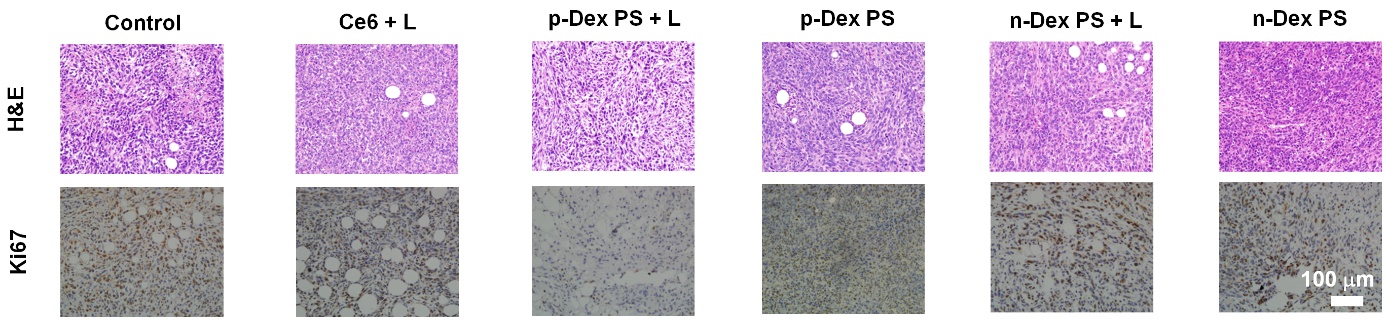


**Figure S9.** H&E and Ki67 staining of tumor slices at 14 days after the treatments.


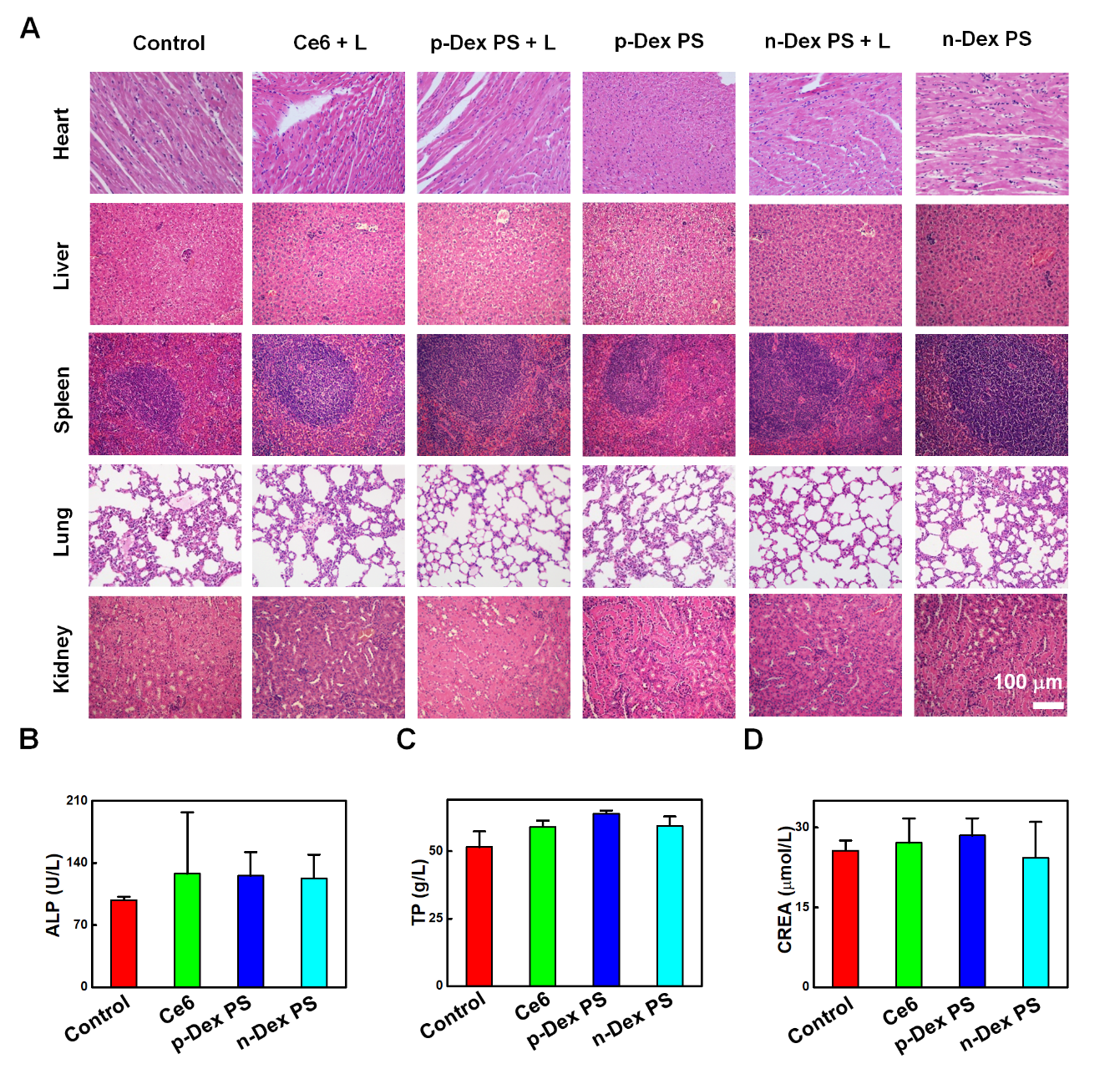


**Figure S10.** (A) H&E staining of major organs obtained from the treated mice at the end of treatments. (B-D) Serum biochemical parameters of 4T1-bearing mice at 2 days after intravenous injection of different formulations.


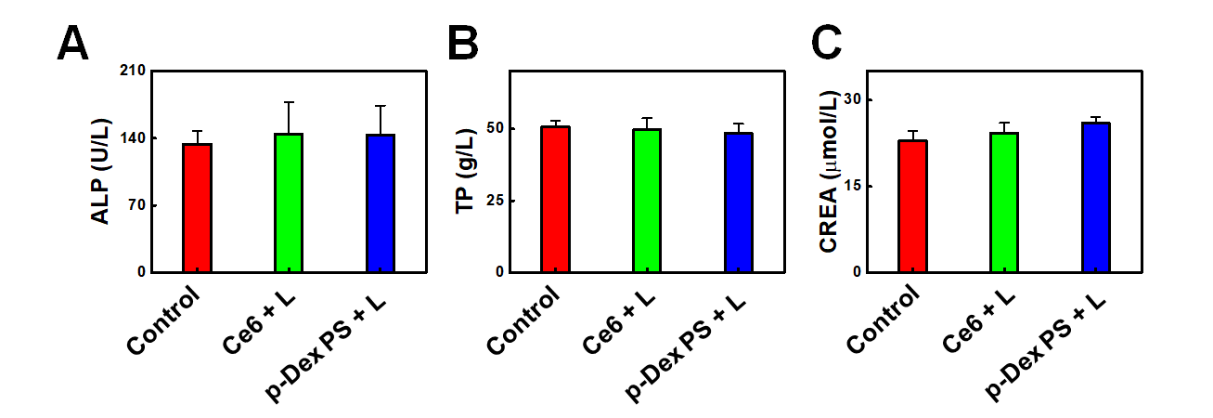


**Figure S11.** Serum biochemical parameters of 4T1-bearing mice at 2 days after intravenous injection of different formulations and the light irradiation of tumor.


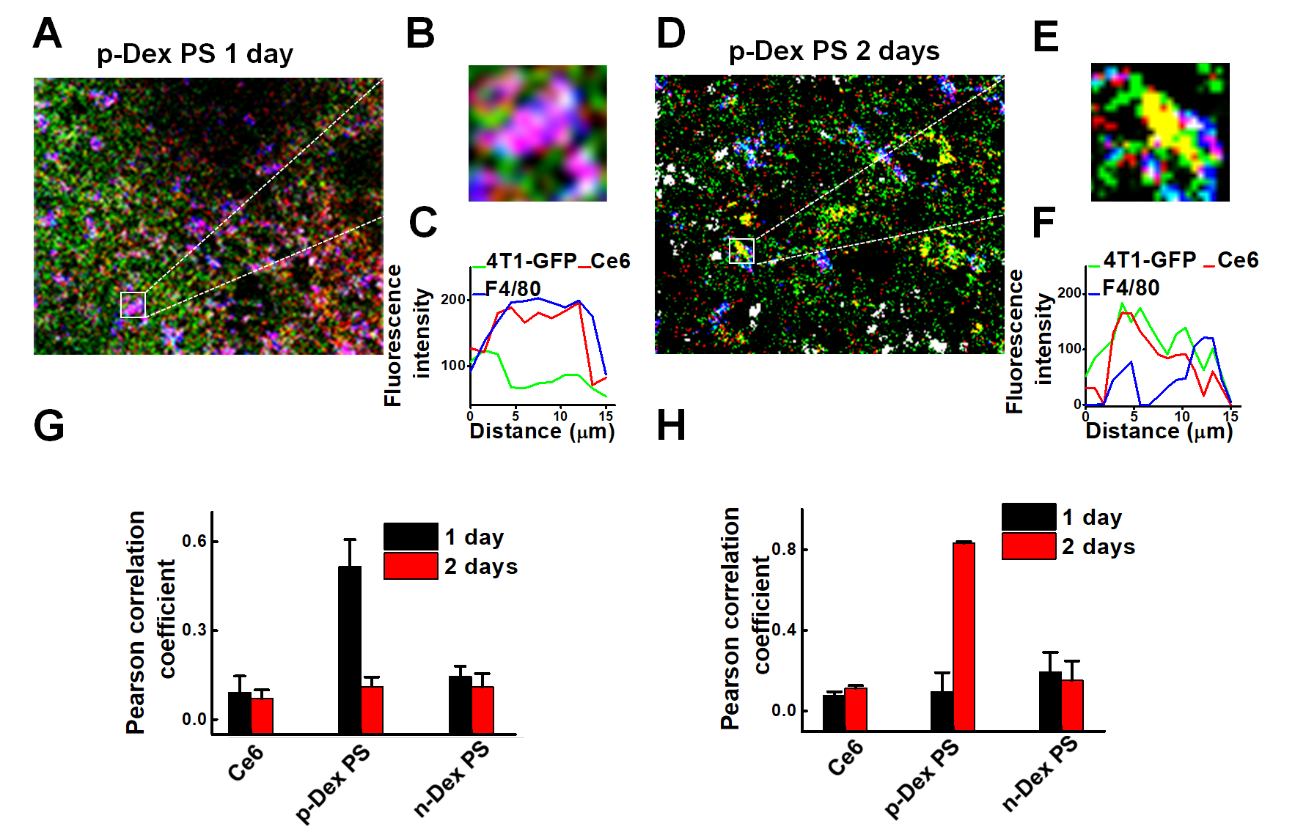


**Figure S12.** Immunofluorescence analysis of tumor frozen sections. The blue, green and red signals represent the fluorescence of F4/80, 4T1-GFP and Ce6, respectively. (A) Representative immunofluorescence image of tumor at 1 day post intravenous injection of p-Dex PS for the colocalizaiton analysis. (B-C) Magnified image region (B) and fluorescence intensity profile of Ce6's colocalization (C) in Figure S12A. (D) Representative immunofluorescence image of tumor at 2 days post intravenous injection of p-Dex PS for the colocalizaiton analysis. (E-F) Magnified image region (E) and representative fluorescence intensity profile of Ce6‘s colocalization (F) in Figure S12D. (G-H) Pearson correlation coefficients representing the colocalization of Ce6 signals with TAMs (G) and tumor cells (H) obtained from Figure 1F.


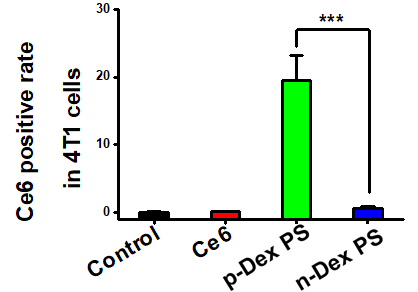


**Figure S13.** Ce6 positive rate in 4T1 cells obtained from the FACS quantification of the direct coincubation of 4T1-GFP cells with RAW264.7 cells. RAW264.7 cells were pre-treated by Ce6, p-Dex Ps and n-Dex PS of equal Ce6 concentrations for 24 h.


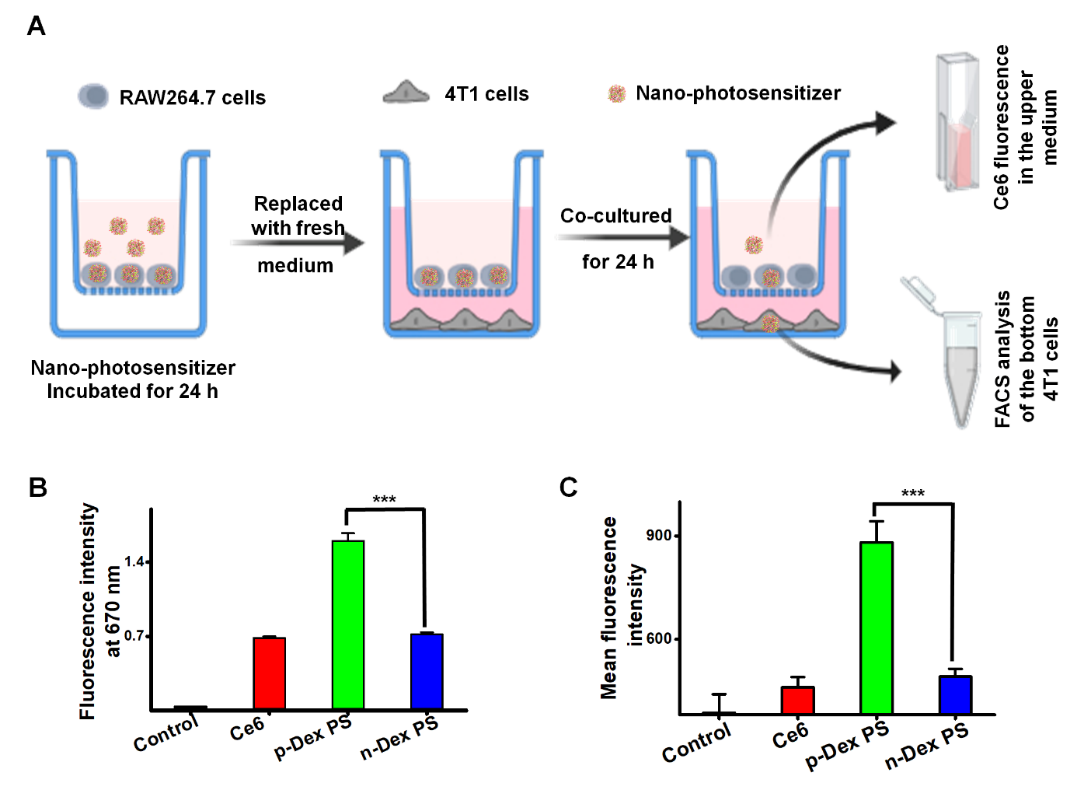


**Figure S14.** (A) Schematic illustration of the intercellular transport and redistribution of nano-photosensitizers in transwell plates. The RAW264.7 cells with different pretreatments were separated from 4T1 cells by the transwell membrane with a pore size of 1 μm. (B) The Ce6 fluorescence intensity at 670 nm in the medium collected from the upper chambers of transwell plates. (C) The FACS analysis of the mean fluorescence intensity of Ce6 in 4T1 cells collected from the bottom chambers of transwell plates.


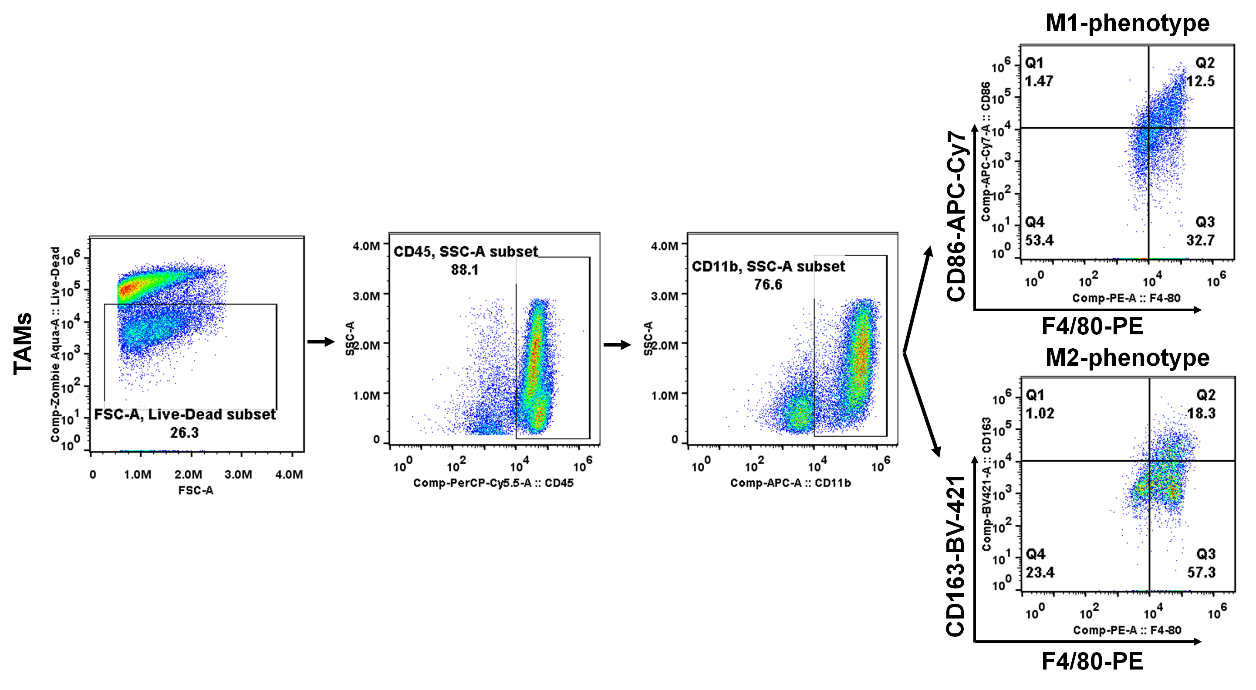


**Figure S15.** FACS gating strategy for M1- and M2-phenotype TAMs.


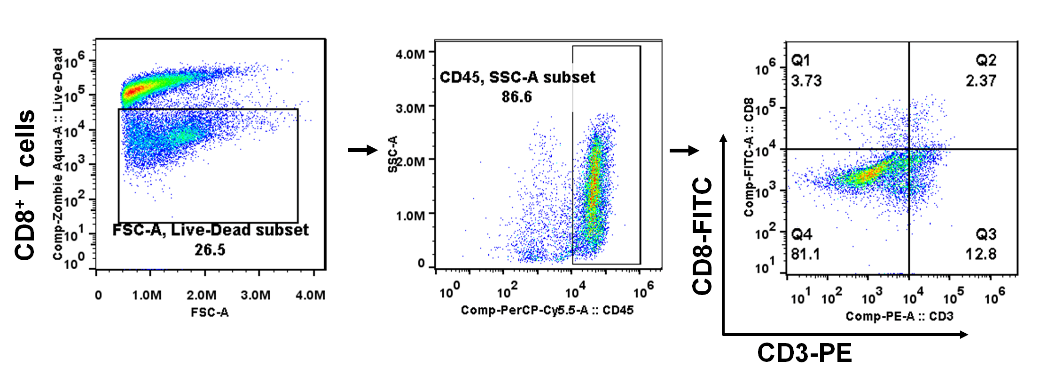


**Figure S16.** FACS gating strategy for CD8^+^ T cells.


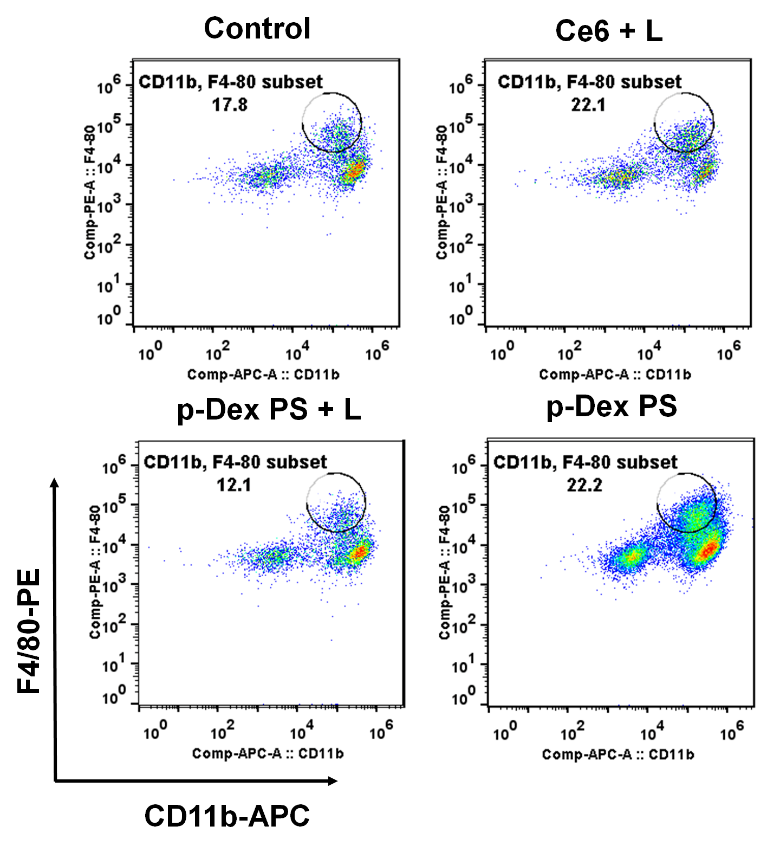


**Figure S17.** FACS analysis of total TAMs in the tumor sites with Control, Ce6+L, p-Dex PS and p-Dex PS+L treatments.


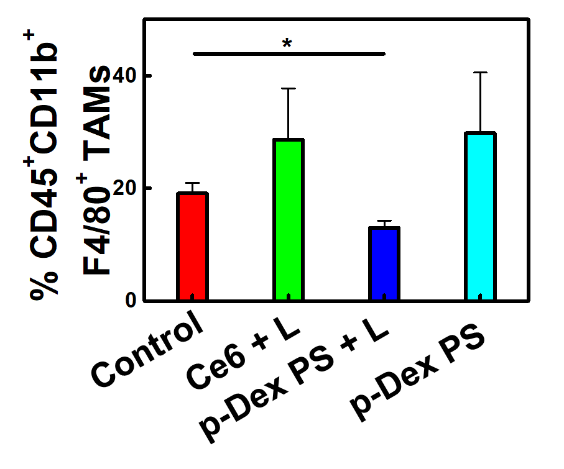


**Figure S18.** Quantitative data of intratumoral TAMs infiltration measured from FACS.


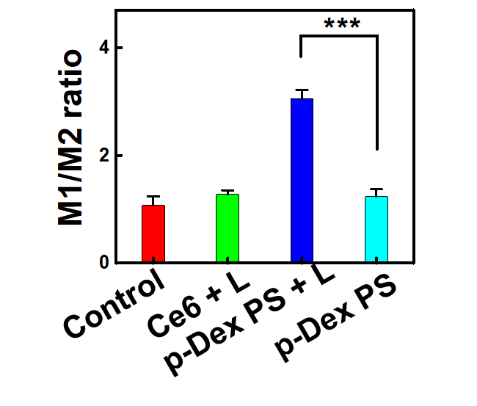


**Figure S19.** Quantitative data of the ratio of intratumoral M1/M2 TAMs measured from FACS.


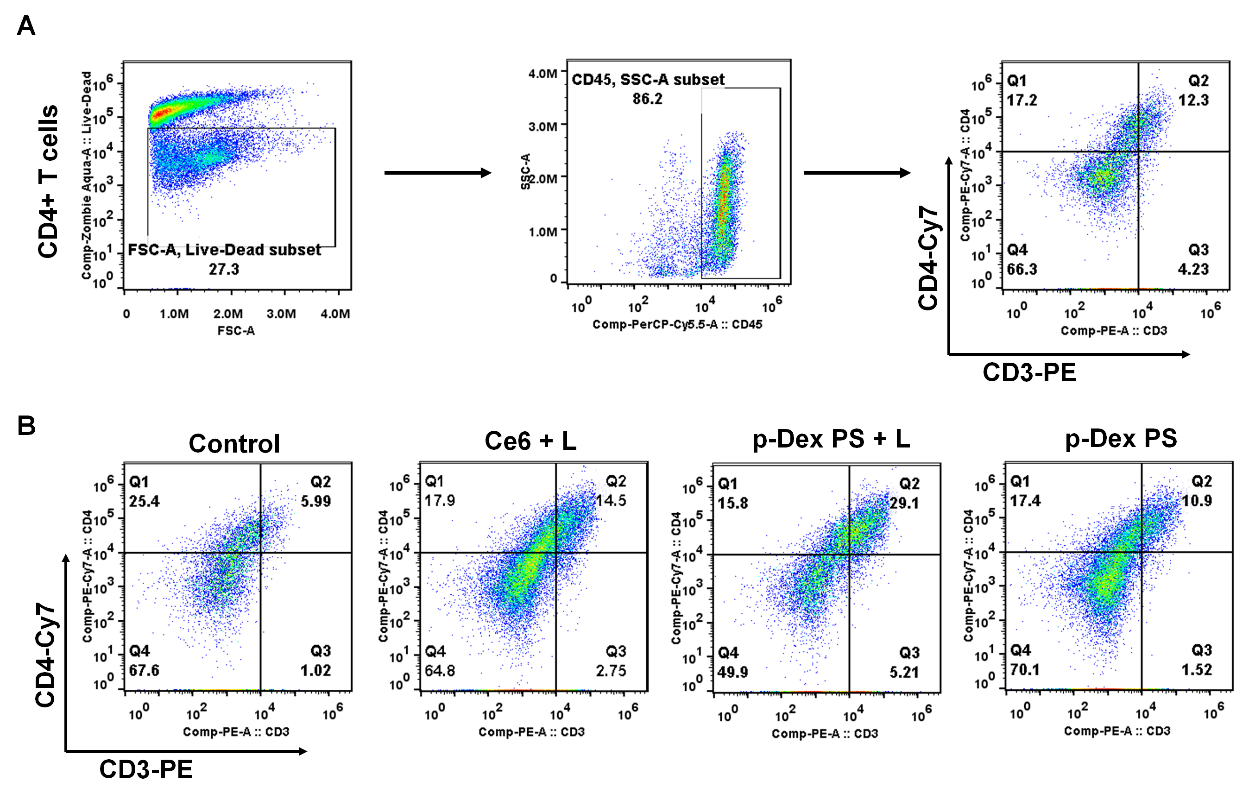


**Figure S20.** (A) FACS gating strategy for CD4^+^ T cells. (B) FACS analysis of CD4^+^ T cells in the control, Ce6+L, p-Dex PS and p-Dex PS+L treated tumor.


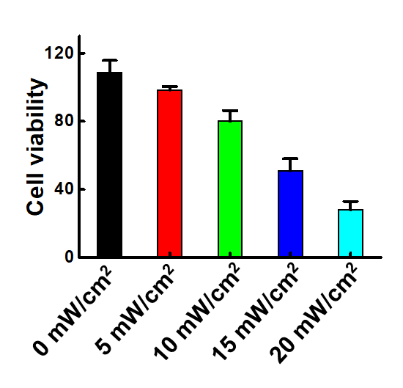


**Figure S21.** The cell viability of RAW264.7 cells treated with p-Dex PS (500 nM Ce6) at different light dose.


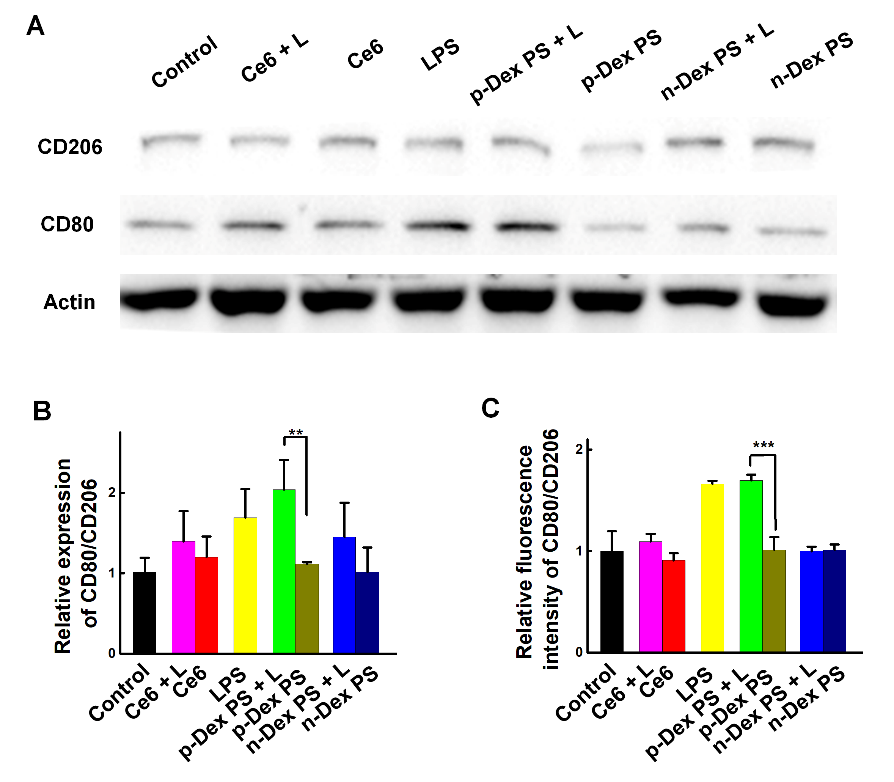


**Figure S22.** (A) The representative images of Western blot analysis of CD80 and CD206 proteins in RAW264.7 cells after treatment with different groups. CD80 and CD206 proteins represent M1 and M2 markers, respectively. (B) Relative expression of CD80/CD206 determined by Western blot. (C) FACS analysis of the ratio of CD80/CD206 in RAW264.7 cells after treatment with different groups.


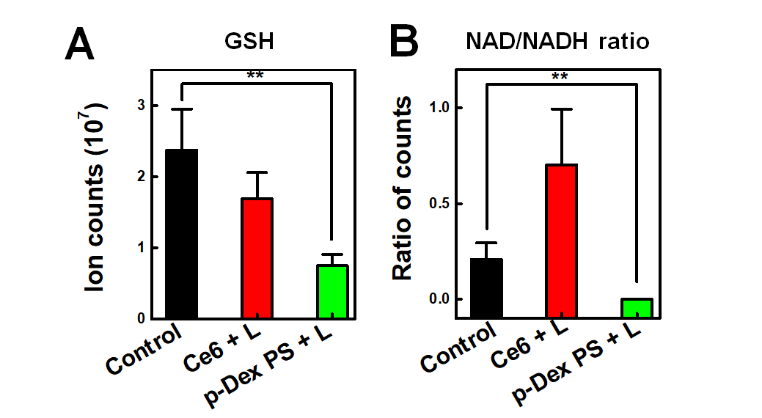


**Figure S23.** GSH and NAD/NADH ratio in RAW264.7 cells after the control, Ce6 PDT and p-Dex PS+L treatments.


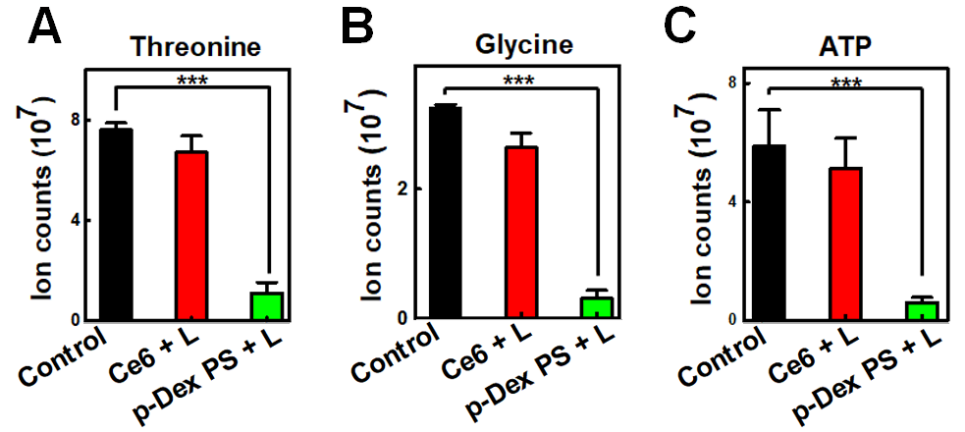


**Figure S24.** Variation of threonine, glycine and ATP in RAW 264.7 cell after the control, Ce6 PDT and p-Dex PS+L treatments.

References

Deng, H., Yang, X., Wang, H.M., Gao, M.H., Zhang, Y.Y., Liu, R.M., Xu, H.Y., and Zhang, W.Q. (2024). Tailoring the surface charges of iron-crosslinked dextran nanogels towards improved tumor-associated macrophage targeting. Carbohydr. Polym. 325, 121585.

Zhu, L.W., You, Y.C., Zhu, M.X., Song, Y.L., Zhang, J.C., Hu, J.H., Xu, X.Y., Xu, X.L., Du, Y.Z., and Ji, J.S. (2022). Ferritin-Hijacking Nanoparticles Spatiotemporally Directing Endogenous Ferroptosis for Synergistic Anticancer Therapy. Adv. Mater. 4, 2207174.
